# A systematic review of the prevalence and correlates of mental and substance use disorders in first nations peoples in Australia, New Zealand, Canada and the United States of America

**DOI:** 10.1007/s00127-026-03104-5

**Published:** 2026-04-29

**Authors:** Tabinda A. Basit, Damian F. Santomauro, Meredith G. Harris, Harvey A. Whiteford, Maree R. Toombs, Megavartini Sivakumar, Alize J. Ferrari

**Affiliations:** 1 School of Psychology, The University of Queensland, St Lucia, QLD 4072 Australia; 2 Queensland Centre for Mental Health Research, Wacol, QLD Australia; 3 School of Public Health, The University of Queensland, Herston, QLD Australia; 4 Institute for Health Metrics and Evaluation, University of Washington, Seattle, USA; 5 School of Public Health, University of Sydney, Sydney, New South Wales Australia

**Keywords:** First Nations people, Mental disorders, Substance use disorders, Prevalence, Correlates, Systematic review

## Abstract

**Purpose:**

First Nations people in Australia, New Zealand, Canada and the United States of America (USA) experience high rates of mental and substance-use disorders (MSDs) and face socially determined health inequalities, but this is not always well quantified. This systematic review sought to summarise best-estimates of MSD prevalence and describe related sociodemographic correlates among First Nations adults in these countries.

**Methods:**

Relevant studies published between 1980 and 2026 were searched using PubMed, Embase, and PsycINFO following PRISMA guidelines. Epidemiological population surveys producing prevalence estimates and/or sociodemographic correlates of MSDs for representative populations of First Nations peoples were identified. Prevalence data and sociodemographic correlates were extracted and described. Meta-analyses were conducted for MSDs with at least three available estimates.

**Results:**

A total of 50 studies from 24 unique population-level representative surveys met inclusion criteria. Surveys from the USA provided the most data (35 studies). Generally, MSD prevalence was lowest in the USA and highest in Australia, with twelve-month rates ranging between 20.9% (18.8–23.1%) and 51.7% (47.3–55.9%) respectively. Mood disorders and anxiety disorders were most prevalent. Sociodemographic variables including sex, socioeconomic factors, and regionality were associated with higher risk of certain MSDs.

**Conclusion:**

The prevalence and related sociodemographic correlates of MSDs identified across countries are varied and have implications for risk profiles and treatment needs. Low numbers of studies and heterogeneous data-collection methodologies highlight the need for better quality, representative population level data on MSDs for First Nations peoples.

**Supplementary Information:**

The online version contains supplementary material available at https://doi.org/10.1007/s00127-026-03104-5.

## Introduction

First Nations people experience poorer health and wellbeing than their non-Indigenous counterparts, even in countries that perform well on measures of development, economic stability and resourcing [1–3]. Australia, New Zealand, Canada and the United States of America (USA) share similar economic circumstances and common histories of colonisation [1, 2], evident in ongoing experiences of subjugation, marginalisation and disrupted connections to identity, health, and wellbeing [4].

First Nations peoples in these countries experience poorer psychological wellbeing and are more likely to access mental health services than the broader population [5–7]. These outcomes are correlated with socio-economic status (SES), education, income, separation from culture or community, and interpersonal violence [8–12]. However, the dearth of representative population surveys in First Nations peoples [13] results in considerably less information on the sociodemographic correlates of specific MSDs. Previous reviews are out of date or focus on subsets of MSDs such as mood and anxiety disorders [14–16]. This makes it difficult for policy makers and service providers to fully quantify mental health needs of First Nations peoples and the most effective service response.

Our systematic review aims to highlight the current state of knowledge about the prevalence and correlates of MSDs in First Nations adults as measured by diagnostic epidemiological surveys and compare these across Australia, New Zealand, Canada and the USA. Given the paucity of available representative data on First Nations populations, this paper intends to provide best-estimate summary metrics to which additional data can be added. It will:

1. Critically appraise data availability and coverage of representative prevalence surveys of MSDs in the identified countries while making recommendations for future research.
2. Summarise best-estimates of MSD prevalence via meta-analysis where possible and describe the reported related sociodemographic correlates among First Nations adults in these countries.
3. Highlight commonalities and differences between the prevalence and sociodemographic correlates of MSDs in First Nations peoples of Australia, New Zealand, Canada, and the USA.

Recommendations to respond to specific data gaps will be discussed.

## Methods

### MSDs and sociodemographic correlates of interest

MSDs were defined by the Diagnostic and Statistical Manual of Mental Disorders (DSM) [17] or International Classification of Diseases (ICD) [18]. Included MSDs were those that have been successfully measured using standardised diagnostic criteria and instruments, falling under broad categories of MSDs. A list of included MSDs is listed in Table 2 and appendix (p. 8). Sociodemographic correlates were guided by existing literature, with most population level surveys also gathering demographic data including sex, age, marital status, socioeconomic measures (income, education level, employment status), and residential regionality.

**Table 1:** List of studies meeting inclusion criteria and their characteristics

Dataset	National/ Regional Survey		Study	Study Design	Sample size	Sample Characteristics	Instrument	Study Quality Appraisal (JBI)	Cultural adaptations
Australia									
N/A	Regional		**Almeida et al.**,** 2014** [54]	Cross-sectional, mixed-methods sampling within First Nations population	235	Age: 46-89 Sex reported: male, female, both	Kimberley Indigenous Cognitive Assessment of Depression (KICA-dep)	High	Culturally validated instrument
N/A	Regional		**Bohanna et al.**,** 2012** [55]	Cross-sectional, snowball sampling method within First Nations population	133	Age: 14-47 Sex reported: both	Severity of Dependence Scale (SDS)	Low	Unclear
Queensland Urban Indigenous Mental Health Survey (QUIMHS)	Regional		**Ferrari et al.**,** 2023** [45]	Cross-sectional, mixed-method sampling within First Nations population	406	Age: 18–99 Sex reported: male, female, both	Composite International Diagnostic Interview (CIDI) 3.0 (DSM-IV-R Criteria)	High	Instrument adapted for use in an Indigenous Australian sample, Culturally informed research methods
			**Basit et al.**,** 2024** [3]		406	Age: 18–99 Sex reported: male, female, both	Composite International Diagnostic Interview (CIDI) 3.0 (DSM-IV-R Criteria)	High	Instrument adapted for use in an Indigenous Australian sample, Culturally informed research methods
Health Omnibus Surveys	Regional		**Burt et al.**,** 2020** [56]	Cross-sectional, random sample	92	Age: 16–99 Sex reported: both	Eating disorder examination (EDE)	High	Unclear
Crossroads II	Regional		**Dashputre et al.**,** 2023** [57]	Cross-sectional, random sample	11	Age: 16–99 Sex reported: both	Patient Health Questionnaire (PHQ-9)	High	Unclear
N/A	Regional		**Nadew et al.**,** 2012** [58]	Cross-sectional, identified sample within First Nations population	221	Age: 18–99 Sex reported: both	Composite International Diagnostic Interview (CIDI) 3.0 (DSM-IV-R Criteria)	Low	Not well described
N/A	Regional		**Nasir et al.**,** 2018** [38]	Cross-sectional, mixed-methods sample within First Nations populations	544	Age: 18–99 Sex reported: male, female, both	Structured Clinical Interview for DSM-IV-R (SCID-I)	High	Culturally informed research methods
N/A	Regional		**Shen et al.**,** 2018** [41]	Cross-sectional, mixed-methods recruitment	336	Age: 60–99 Sex reported: both	Modified Patient Health Questionnaire (mPHQ)	High	Culturally validated instrument, Culturally informed research methods
Grog Survey App	Regional		**Weatherall et al.**,** 2022** [59]	Cross-sectional, convenience sample	775	Age: 16–99 Sex reported: both	Questions developed and based on ICD-11 criteria	High	Instrument developed for use in an Indigenous Australian sample, Culturally informed research methods
New Zealand									
Te Rau Hinengaro: The New Zealand Mental Health Survey	National		**Baxter et al.**,** 2006** [12]	Cross-sectional, random sample, First Nations people oversampled relative to proportions within the population	2595	Age: 16-99 Sex reported: both	Composite International Diagnostic Interview (CIDI) 3.0	High	Culturally informed study methods
Canada									
N/A	Regional		**Froese et al.**,** 2008** [60]	Cross-sectional, random sample	430	Age: 18–99 Sex reported: female	Patient Health Questionnaire (PHQ-9)	High	Unclear
Canadian Community Health Survey-Mental Health (CCHS-MH)	National		**Fuller-Thomson et al.**,** 2020** [61]	Cross-sectional, multi-stage cluster sampling design with random sampling	965	Age: 15-99 Sex reported: both	Composite International Diagnostic Interview (CIDI) 3.0	High	Unclear
Ontario First Nations Regional Health Survey (OFNRHS)	Regional		**MacMillan et al.**,** 2008** [62]	Cross-sectional, stratified random sample within identified reservations	769	Age: 18–44 Sex reported: female	Composite International Diagnostic Interview (CIDI) 3.0	High	Unclear
Nunavik Inuit Health Survey	Regional		**Muckle et al.**,** 2017** [40]	Cross-sectional, stratified random sample, oversampled relative to proportions within the population	1326	Age: 16–99 Sex reported: male, female, both	Center for Epidemiological Studies Depression (CESD)	High	Culturally validated instrument, Culturally informed research methods
United States of America									
The American Indian Service Utilization, Psychiatric Epidemiology, Risk and Protective Factors Project (AI-SUPERPFP)	Regional		**Beals et al.**,** 2005a** [31]	Cross-sectional, stratified random sample within identified reservations	3084	Age: 15–57 Sex reported: male, female, both	Composite International Diagnostic Interview (CIDI) 3.0 (DSM-IV Criteria)	High	Instrument adapted for use in American Indian communities in the context of a previous study
			**Beals et al.**,** 2005b** [63]		3084	Age: 15–57 Sex reported: male, female, both	Composite International Diagnostic Interview (CIDI) 3.0 (DSM-III Criteria)	High	Instrument adapted for use in American Indian communities in the context of a previous study
			**Beals et al.**,** 2013** [64]		1697	Age: 15–57 Sex reported: male, female, both	Composite International Diagnostic Interview (CIDI) 3.0 (DSM-III Criteria)	High	Instrument adapted for use in American Indian communities in the context of a previous study
			**Sawchuk et al.**,** 2005** [65]		1414	Age: 18–57 Sex reported: both	Composite International Diagnostic Interview (CIDI) 3.0 (DSM-III Criteria)	High	Instrument adapted for use in American Indian communities in the context of a previous study
National Epidemiologic Survey on Alcohol and Related Conditions	National		**Brave Heart et al.**,** 2016** [66]	Cross-sectional, random sample	701	Age: 18–99 Sex reported: male, female, both	NIAAA Alcohol Use Disorder and Associated Disabilities Interview Schedule-DSM-IV Version (AUDADIS-IV)	High	Unclear
			**Grant et al.**,** 2004** [67]		701	Age: 18–99 Sex reported: both	NIAAA Alcohol Use Disorder and Associated Disabilities Interview Schedule-DSM-IV Version (AUDADIS-IV)	High	Unclear
			**Grant et al.**,** 2005a** [68]		701	Age: 24–99 Sex reported: both	NIAAA Alcohol Use Disorder and Associated Disabilities Interview Schedule-DSM-IV Version (AUDADIS-IV)	High	Unclear
			**Grant et al.**,** 2005b** [69]		701	Age: 18–99 Sex reported: both	NIAAA Alcohol Use Disorder and Associated Disabilities Interview Schedule-DSM-IV Version (AUDADIS-IV)	High	Unclear
			**Grant et al.**,** 2006** [70]		701	Age: 18–99 Sex reported: both	NIAAA Alcohol Use Disorder and Associated Disabilities Interview Schedule-DSM-IV Version (AUDADIS-IV)	High	Unclear
			**Grant et al.**,** 2008** [71]		701	Age: 18–99 Sex reported: male, female, both	NIAAA Alcohol Use Disorder and Associated Disabilities Interview Schedule-DSM-IV Version (AUDADIS-IV)	High	Unclear
			**Grant et al.**,** 2015** [72]		511	Age: 18–99 Sex reported: both	NIAAA Alcohol Use Disorder and Associated Disabilities Interview Schedule (AUDADIS-5)	High	Unclear
			**Grant et al.**,** 2016** [73]		511	Age: 18–99 Sex reported: both	NIAAA Alcohol Use Disorder and Associated Disabilities Interview Schedule (AUDADIS-5)	High	Unclear
			**Hasin et al.**,** 2005** [74]		511	Age: 18–99 Sex reported: both	NIAAA Alcohol Use Disorder and Associated Disabilities Interview Schedule-DSM-IV Version (AUDADIS-IV)	High	Unclear
			**Hasin et al.**,** 2007** [75]		701	Age: 18–99 Sex reported: both	NIAAA Alcohol Use Disorder and Associated Disabilities Interview Schedule-DSM-IV Version (AUDADIS-IV)	High	Unclear
			**Hasin et al.**,** 2016** [76]		511	Age: 18–99 Sex reported: both	NIAAA Alcohol Use Disorder and Associated Disabilities Interview Schedule-DSM-IV Version (AUDADIS-IV)	High	Unclear
			**Huang et al.**,** 2006** [77]		701	Age: 26–99 Sex reported: both	NIAAA Alcohol Use Disorder and Associated Disabilities Interview Schedule-DSM-IV Version (AUDADIS-IV)	High	Unclear
			**Kerridge et al.**,** 2015** [78]		1022	Age: 18–99 Sex reported: male, female, both	NIAAA Alcohol Use Disorder and Associated Disabilities Interview Schedule (AUDADIS-5)	High	Unclear
			**Kerridge et al.**,** 2018** [79]		1022	Age: 18–99 Sex reported: male, female, both	NIAAA Alcohol Use Disorder and Associated Disabilities Interview Schedule (AUDADIS-5)	High	Unclear
			**Pulay et al.**,** 2009** [80]		701	Age: 26–99 Sex reported: male, female, both	NIAAA Alcohol Use Disorder and Associated Disabilities Interview Schedule-DSM-IV Version (AUDADIS-IV)	High	Unclear
			**Saha et al.**,** 2016** [81]		511	Age: 18–99 Sex reported: both	NIAAA Alcohol Use Disorder and Associated Disabilities Interview Schedule (AUDADIS-5)	High	Unclear
			**Smith et al.**,** 2006** [82]		701	Age: 21–99 Sex reported: both	NIAAA Alcohol Use Disorder and Associated Disabilities Interview Schedule-DSM-IV Version (AUDADIS-IV)	High	Unclear
			**Stinson et al.**,** 2006** [83]		701	Age: 18–99 Sex reported: both	NIAAA Alcohol Use Disorder and Associated Disabilities Interview Schedule-DSM-IV Version (AUDADIS-IV)	High	Unclear
			**Stinson et al.**,** 2008** [84]		701	Age: 26–99 Sex reported: male, female, both	NIAAA Alcohol Use Disorder and Associated Disabilities Interview Schedule-DSM-IV Version (AUDADIS-IV)	High	Unclear
National Epidemiologic Survey on Alcohol and Related Conditions – III			**Blanco et al.**,** 2017** [85]	Cross-sectional, random sample	511	Age: 18–99 Sex reported: both	NIAAA Alcohol Use Disorder and Associated Disabilities Interview Schedule (AUDADIS-5)	High	Unclear
			**Emerson et al.**,** 2017** [86]		511	Age: 18–99 Sex reported: both	NIAAA Alcohol Use Disorder and Associated Disabilities Interview Schedule (AUDADIS-5)	High	Unclear
			**Goldstein et al.**,** 2016** [87]		511	Age: 18–99 Sex reported: both	NIAAA Alcohol Use Disorder and Associated Disabilities Interview Schedule (AUDADIS-5)	High	Unclear
			**Hasin et al.**,** 2018** [88]		511	Age: 18–99 Sex reported: both	NIAAA Alcohol Use Disorder and Associated Disabilities Interview Schedule-DSM-IV Version (AUDADIS-IV)	High	Unclear
Intramural Program of the National Institute on Alcohol Abuse and Alcoholism			**Robin et al.**,** 1997** [89]	Cross-sectional, random sample within identified tribes	247	Age: 21–99 Sex reported: male, female, both	Structured Clinical Interview for DSM-III-R (SCID)	High	Culturally informed research methods
			**Robin et al.**,** 2007** [90]	Cross-sectional, random sample within identified tribes	913	Age: 21–99 Sex reported: male, female, both	Structured Clinical Interview for DSM-III-R (SCID)	High	Culturally informed research methods
Long-Term Care and Social Support: American Indian Aged project	Regional		**Curyto et al.**,** 1998** [39]	Longitudinal (first wave), random sample within identified tribes	301	Age: 55–99 Sex reported: both	Center for Epidemiological Studies Depression (CESD)	High	Culturally validated instrument
The Alaska Education and Research Towards Health (EARTH) Study	Regional		**Dillard et al.**,** 2012** [91]	Cross-sectional, snowball sampling method within First Nations population	3771	Age: 18–99 Sex reported: male, female, both	Patient Health Questionnaire (PHQ-9)	High	Instrument adapted for use in American Indian communities, Culturally informed study methods
Ten Tribes Study	National		**Koss et al.**,** 2003** [92]	Cross-sectional, random sample within identified tribes	1670	Age: 20–81 Sex reported: male, female, both	NIAAA Alcohol Use Disorder and Associated Disabilities Interview Schedule-DSM-IV Version (AUDADIS-IV)	High	Instrument adapted for use in American Indian communities
National Institute of Diabetes and Digestive and Kidney Diseases (NIDDK)	Regional		**Sahota et al.**,** 2008** [93]	Longitudinal (first wave), random sample within identified community	2902	Age: 18–45 Sex reported: male, female, both	Patient Health Questionnaire (PHQ-9)	High	Not well described
Strong Heart Family Study (SHFS)	Regional		**Zhao et al.**,** 2016** [94]	Cross-sectional, First Nations subset within broader study	2175	Age: 40.4 (mean) Sex reported: male, female, both	Center for Epidemiological Studies Depression (CESD)	High	Unclear
N/A	Regional		**Kinzie et al.**,** 1992** [95]	Cross-sectional, random sample within identified community	131	Age: 20–99 Sex reported: male, female, both	Schedule for Affective Disorders and Schizophrenia-Lifetime version (SADS-L)	High	Not well described

**Table 2:** Prevalence estimates for MSDs using diagnostic data across Australia, New Zealand, Canada and the United States of America

Mental or Substance Use Disorder	Prevalence Type	Australia			New Zealand			Canada			United States of America		
		Prevalence (%)	CI (95%)	Number of studies	Prevalence (%)	CI (95%)	Number of studies	Prevalence (%)	CI (95%)	Number of studies	Prevalence (%)	CI (95%)	Number of studies
Any mental disorder	Point	45.4	41.1–49.7	1	18.3	16.2–20.6	1	-	-	-	-	-	-
	12-month	40.2–51.7^*b*^	35.2–45.4 47.3–55.9	2	29.5	26.7–32.5	1	-	-	-	20.9–24.2^*b*^	18.8–23.1 22.1–26.3	1 (2 datapoints)
	Lifetime	66.9	62.7–70.8	1	50.7	47.0–54.4	1	-	-	-	41.8–44.4^*b*^	39.2–44.4 41.9–46.8	1 (2 datapoints)
Any mood disorder	Point	18	14.8–21.5	1	4.1	3.3–5.1	1	-	-	-	-	-	-
	12-month	21.5	18.1–25.2	1	11.4	10.0–13.1	1	-	-	-	14.3 -15.4^*b*^	11.8–17.1 12.8–18.3	2
	Lifetime	36.2	32.1–40.4	1	24.3	22.4–26.3	1	-	-	-	28.6	25.4–32.2	1
Any depressive disorder	Point	7.7	4.3–11.1	1	-	-	-	-	-	-	-	-	-
	12-month	-	-	-	-	-	-	-	-	-	4.6–7.3^*b*^	3.7–5.8 6.8–8.8	1 (2 datapoints)
	Lifetime	-	-	-	-	-	-	-	-	-	8.1–11.3^*b*^	6.8–9.6 9.7–13.0	1 (2 datapoints)
Major Depressive Disorder	Point	10.3–45.5^*b*^	16.7–76.6 7.8–13.1	2	-	-	-	-	-	-	-	-	-
	12-month	13.1–24.6^*b*^	10.3–16.1 20.4–29.3	2	6.9	5.8–8.1	1	-	-	-	7.6^*c*^	4.5–11.3	3 (4 datapoints)
	Lifetime	20.0–23.9^*b*^	14.8–25.7 20.3–27.7	2	15.7	14.2–17.4	1	27.1	24.3–29.9	1	13.7^*c*^	8.2–20.2	4 (5 datapoints)
Dysthymia	Point	2.8	1.5–4.5	1	-	-	-	-	-	-	-	-	-
	12-month	2.8	1.5–4.5	1	1.2	0.8–1.7	1	-	-	-	1.8^*c*^	0.8–3.2	2 (3 datapoints)
	Lifetime	1.8–3.1^*b*^	0.5–4.5 1.8–4.9	2	2.1	1.5–2.8	1	-	-	-	3.5^*c*^	2.2–5.2	2 (3 datapoints)
Bipolar Disorder	Point	1.7	0.7–3.1	1	-	-	-	-	-	-	-	-	-
	12-month	1.6	0.7–3.1	1	4.6	3.7–5.7	1	-	-	-	3.9–4.5 ^*b*^	3.5 − 7.7 3.1–6.4	2
	Lifetime	2.4	1.2–4.1	1	8.3	7.1–9.7	1	30.6	27.7–33.5	1	5.6–8.3 ^*b*^	2.1–5.7 6.3–10.6	2
Mania	12-month	-	-	-	-	-	-	-	-	-	2.5	1.5–4.0	1
Any anxiety disorder	Point	31.3	31.3	1	13.4	11.6–15.4	1	-	-	-	-	-	-
	12-month	34.2	34.2	1	19.4	17.2–21.8	1	-	-	-	9.4^*c*^	5.6–14.0	2 (3 datapoints)
	Lifetime	17.2–42.8^*b*^	12.4 -22.8 38.6–47.1	2	31.3	28.4–34.3	1	-	-	-	19.9^*c*^	15.2–24.9	2 (3 datapoints)
Generalised Anxiety Disorder	Point	5.3	3.6–7.5	1	-	-	-	-	-	-	-	-	-
	12-month	5.9–8.1^*b*^	4.0–8.2 5.7–11.4	2	2.2	1.6–2.9	1	-	-	-	1.6^*c*^	0.1–2.7	2 (3 datapoints)
	Lifetime	6.1–4.5^*b*^	4.2–8.4 2.1–8.1	2	5.9	4.9–7.0	1	20.8	18.2–23.4	1	4.8^*c*^	1.8–9.0	3 (4 datapoints)
Panic Disorder	Point	5.8	4.0–8.2	1	-	-	-	-	-	-	-	-	-
	12-month	8.5	6.2–11.1	1	2.6	2.0–3.5	1	-	-	-	2.4^*c*^	1.0–4.4	2 (3 datapoints)
	Lifetime	1.3–12.1^*b*^	0.2–3.9 9.5–15.1	2	3.9	3.1–4.9	1	-	-	-	4.8^*c*^	1.9–8.8	2 (3 datapoints)
Post-Traumatic Stress Disorder	Point	13.8	11.0–16.9	1	-	-	-	-	-	-	5.7	3.1–9.3	1
	12-month	15.3–19.9^*b*^	12.3–18.5 16.1–24.5	2	4.5	3.6–5.7	1	-	-	-	7.2^*c*^	3.9–11.3	2 (3 datapoints)
	Lifetime	20.8	17.4–24.4	1	9.7	8.2–11.4	1	-	-	-	15.2^*c*^	12.2–18.4	4 (5 datapoints)
Social anxiety disorder	Point	7.7	5.6–10.2	1	-	-	-	-	-	-	-	-	-
	12-month	8.3	6.1–10.9	1	6.2	5.1–7.4	1	-	-	-	3.6	2.3–5.6	1
	Lifetime	1.8–11.2^*b*^	0.5–4.5 8.6–14.1	2	11.4	9.9–13.0	1	-	-	-	8.6	6.6–10.9	1
Agoraphobia	12-month	-	-	-	1	0.6–1.5	1	-	-	-	-	-	-
	Lifetime	-	-	-	1.8	1.2–2.7	1	-	-	-	-	-	-
Specific Phobia	Point	9.9	7.5–12.7	1	-	-	-	-	-	-	-	-	-
	12-month	10.1	7.7–12.9	1	11	9.6–12.6	1	-	-	-	8.2	6.3–10.6	1
	Lifetime	8.1–12.1^*b*^	4.9–12.5 9.5–15.1	2	15.3	13.7–17.1	1	-	-	-	12.2	9.3–14.9	1
Schizophrenia	Lifetime	-	-	-	-	-	-	-	-	-	0.3–0.9^*b*^	0.0–1.6 0.0–1.9	1 (2 datapoints)
Obsessive Compulsive Disorder	Point	2.4	1.2–4	1	-	-	-	-	-	-	-	-	-
	12-month	2.6	1.4–4.2	1	1	0.6–1.6	1	-	-	-	-	-	-
	Lifetime	2.9	1.6–4.7	1	2.6	1.8–3.7	1	-	-	-	-	-	-
Any eating disorder	Point	-	-	-	0.5	0.2–1.0	1	-	-	-	-	-	-
	12-month	-	-	-	1	0.5–1.6	1	-	-	-	-	-	-
	Lifetime	-	-	-	3.1	2.3–4.1	1	-	-	-	-	-	-
Anorexia	12-month	-	-	-	0	0.0–0.0	1	-	-	-	-	-	-
	Lifetime	-	-	-	0.7	0.2–1.6	1	-	-	-	-	-	-
Bulimia	12-month	-	-	-	1	0.5–1.6	1	-	-	-	-	-	-
	Lifetime	-	-	-	2.4	1.8–3.2	1	-	-	-	-	-	-
Substance Use Disorders													
Any substance use disorder	Point	13.4	10.6–16.5	1	-	-	-	-	-	-	-	-	-
	12-month	18.8	15.6–22.2	1	8.6	7.1–10.4	1	-	-	-	9.2^*c*^	4.5–15.2	3 (4 datapoints)
	Lifetime	41.5	37.7–45.8	1	26.5	24.3–28.7	1	-	-	-	26.4^*c*^	16.9–37.1	2 (3 datapoints)
Substance Abuse	Point	2	1–3.5	1	-	-	-	-	-	-	-	-	-
	12-month	3.3	1.9–5.1	1	3.7	2.8–4.8	1	-	-	-	2.3^*c*^	1.4–3.3	2 (3 datapoints)
	Lifetime	8.6	6.4–11.3	1	14.3	12.6–16.1	1	-	-	-	5.1–8.3^*b*^	3.9–6.3 7.0–9.8	1 (2 datapoints)
Substance Dependence	Point	3.1	1.8–4.9	1	-	-	-	-	-	-	-	-	-
	12-month	4.8	3.1–6.9	1	1.9	1.3–2.8	1	-	-	-	2.5^*c*^	2.0–3.0	2 (3 datapoints)
	Lifetime	11.2	8.6–14.1	1	6.3	5.2–7.6	1	28.8	26.0–31.6	1	3.9–4.8^*b*^	3.0–5.1 3.8–5.9	1 (2 datapoints)
Any alcohol use disorder	12-month	-	-	-	7.4	6.2–8.9	1	-	-	-	12.1–19.2^*b*^	9.8–14.8 15.8 -22.9	2
	Lifetime	-	-	-	24.5	22.3–26.7	1	39.8	36.7–42.9	1	43.0–43.4^*b*^	39.2–46.7 39.1–47.9	2
Alcohol Abuse	Point	4.4	2.8–6.4	1	-	-	-	-	-	-	-	-	-
	12-month	7.4	5.3–9.8	1	6.7	5.5–8.1	1	-	-	-	5.4^*c*^	3.8–7.2	2 (3 datapoints)
	Lifetime	19.3	16–22.8	1	24.4	22.3–26.7	1	-	-	-	14.4^*c*^	8.2–2.2	3 (4 datapoints)
Alcohol Dependence	Point	6.6	4.6–9	1	-	-	-	-	-	-	-	-	-
	12-month	2.2–9.9^*b*^	1.2–3.4 7.5–12.7	2	3.9	3.0–5.0	1	-	-	-	6.5^*c*^	3.5–10.4	2 (3 datapoints)
	Lifetime	22.9	19.5–26.7	1	10.1	8.7–11.7	1	-	-	-	16.7^*c*^	11.0–23.4	3 (4 datapoints)
Any cannabis use disorder	12-month	-	-	-	-	-	-	-	-	-	3.4–5.3^*b*^	2.2–5.1 3.5–7.6	2
	Lifetime	-	-	-	-	-	-	-	-	-	11.5–15.4^*b*^	8.9–14.6 12.8–18.3	2
Cannabis Abuse	12-month	-	-	-	3	2.3–4.0	1	-	-	-	2.1	1.2–3.5	1
	Lifetime	-	-	-	12.8	11.2–14.6	1	-	-	-	12.3	9.9–14.9	1
	12-month	-	-	-	1.5	1.0–2.3	1	-	-	-	1.3	0.1–2.4	1
Cannabis Dependence													
	Lifetime	-	-	-	5.3	4.3–6.5	1	38.7	35.6–41.8	1	3.1	1.9–4.7	1
Prescription Opioid Use Disorder	12-month	-	-	-	-	-	-	-	-	-	1.4–1.6^*b*^	0.1–2.8 0.9–2.7	2
	Lifetime	-	-	-	-	-	-	-	-	-	4.1^*c*^	3.4–5.0	3
Nicotine Dependence	12-month	-	-	-	-	-	-	-	-	-	23.0	19.9–26.3	1
	Lifetime	-	-	-	-	-	-	-	-	-	30.0	26.6–33.5	1
Personality Disorders													
Any personality disorder	Lifetime	-	-	-	-	-	-	-	-	-	24.0	20.8–27.3	1
Avoidant personality disorder	Lifetime	-	-	-	-	-	-	-	-	-	3.8	2.4–5.4	1
Dependent personality disorder	Lifetime	-	-	-	-	-	-	-	-	-	0.5	0.2–1.5	1
Histrionic personality disorder	Lifetime	-	-	-	-	-	-	-	-	-	2.4	1.4–3.8	1
Obsessive-compulsive personality disorder	Lifetime	-	-	-	-	-	-	-	-	-	9.9	7.7–12.3	1
Paranoid personality disorder	Lifetime	-	-	-	-	-	-	-	-	-	10.6	8.8–13.1	1
Schizoid personality disorder	Lifetime	-	-	-	-	-	-	-	-	-	6.2	4.5–8.2	1
Borderline personality disorder	Lifetime	-	-	-	-	-	-	-	-	-	11.9	9.5–14.3	1
Schizotypal personality disorder	Lifetime	-	-	-	-	-	-	-	-	-	6.6	4.8–8.4	1
Narcissistic personality disorder	Lifetime	-	-	-	-	-	-	-	-	-	7.1	5.2–9.0	1

Note: Only available estimates are provided. Single estimates (where only one estimate was available, ^b^Ranges (where there were two estimates reported), ^c^Pooled prevalence estimates (where there were three or more estimates reported)

### Search strategy

The systematic literature review was registered with the International Prospective Register of Systematic Reviews (PROSPERO) [19] (PROSPERO, CRD42021239179). The search string was revised prior to final analysis to prioritise data on prevalence and re-run to include the most recent available evidence; no other aspects of the registered protocol were modified. The search included a review of electronic and grey literature and adhered to guidelines specified by the Preferred Reporting Items for Systematic Review and Meta-Analysis (PRISMA) framework [20]. Three scholarly databases (PubMed, EMBASE and PsycINFO) were searched between January 1980 (to align with the first publication of DSM classifications) to March 2026. No other limits were utilised. A grey literature search involved online searches of government and non-government organisations focused on population health statistics, First Nations health and wellbeing or health parity. This followed an established framework for searching grey literature for epidemiological data on mental disorders [21]. A snowball search involved discussions with experts in the field and additionally reviewed reference lists from review articles. (See appendix p. 3–5 for more details on search methodology).

### Inclusion and exclusion

Epidemiological surveys estimating the prevalence of MSDs amongst First Nations adults in Australia, New Zealand, Canada or the USA were included. Studies broadly adhering to MSDs as defined in DSM/ICD, including surveys using instruments fully adhering to DSM/ICD clinical criteria, as well as symptom scales that partly adhere to these criteria were included. Studies were excluded if estimates produced were of non-specific psychological distress, general emotional health and/or wellbeing.

Both cross-sectional and longitudinal surveys were accepted. Studies producing a sample representative of the First Nations population of that geographic region were included. This included population surveys producing separate estimates for First Nations subgroups, and studies focusing on First Nations peoples. Studies were excluded if the sample was characterised by increased or decreased risk of MSDs, or where systematic bias affected the representativeness of the sample relative to the broader population (for example, incarcerated or clinical samples).

### Data review, extraction, and cleaning

Citations were downloaded and duplicates removed. Titles and abstracts were screened independently for inclusion by two reviewers (TAB, MS). Records not meeting inclusion criteria were excluded. Full-text screening of all potentially eligible records was conducted by both reviewers. Where questions or disagreements arose at the full-text stage, these were discussed with a senior researcher (AJF), and final inclusion decisions were made by consensus. Data extraction was conducted by one reviewer (TAB) using a standardised extraction form. All extracted data and final inclusion decisions were reviewed by a senior researcher (AJF). Any uncertainties were resolved through discussion and consensus. For all surveys meeting inclusion criteria, information related to the publication, sample demographics, data collection, and survey outputs, (i.e., prevalence estimates and measures of correlation) were extracted. For studies also reporting on correlates, odds-ratios or prevalence estimates across levels of a given correlate were extracted. The 95% confidence intervals around the mean prevalence were extracted if reported, or calculated using S.E.= √1.96 (P (1 – P)/N), where P is the proportion of cases reported, and N is the sample size. Male and female estimates were aggregated into an additional estimate for both sexes where possible to maximise opportunities for analysis. More detail on data extraction and cleaning is reported in the appendix (p.6).

### Quality appraisal

Study quality was assessed using the Joanna Briggs Institute (JBI) Critical Appraisal Checklist for Studies Reporting Prevalence Data. This approach was selected due to the limited applicability of conventional risk-of-bias tools for population-based diagnostic epidemiological studies [22]. Each study was assigned an overall rating (low, moderate, or high) based on methodological quality. Summary ratings are presented in Table 1, with full assessments provided in the appendix (p.11).

### Data analysis

MSD prevalence: Data extracted from surveys using diagnostic instruments was analysed/reported by MSD, sex, and recall period (point/current, 12-month, lifetime) as these are known sources of variation in prevalence [23]. Single estimates were presented as is, MSDs with two estimates were presented as a range. For MSDs with three or more estimates, a meta-analysis tool, MetaXL, Version 5.3 [24] was used to pool proportions. A random effects model was chosen over a fixed effects model to better account for heterogeneity between estimates across studies [25, 26]. Heterogeneity was assessed using Q-test and I^2^. All studies included in meta-analyses were screened for outliers and each outlier examined for possible explanations (e.g., differing methodology, age group or diagnostic cut-off score. See appendix p.6 for examples). If there was no readily apparent explanation, that study was retained in that meta-analysis. Forest plots and tests for publication bias were generated (see appendix p. 20–55). Data extracted from symptom scales reported on separately. These scales typically over-estimate the prevalence of MSDs as they identify individuals with symptoms of a given MSD and are not limited to those meeting full diagnostic criteria [23].

Sociodemographic correlates of MSDs: Due to heterogeneity of the available extracted data and the small number of studies available, the review of correlate data was descriptive only.

## Results

### Data availability and coverage

**Fig. 1:**
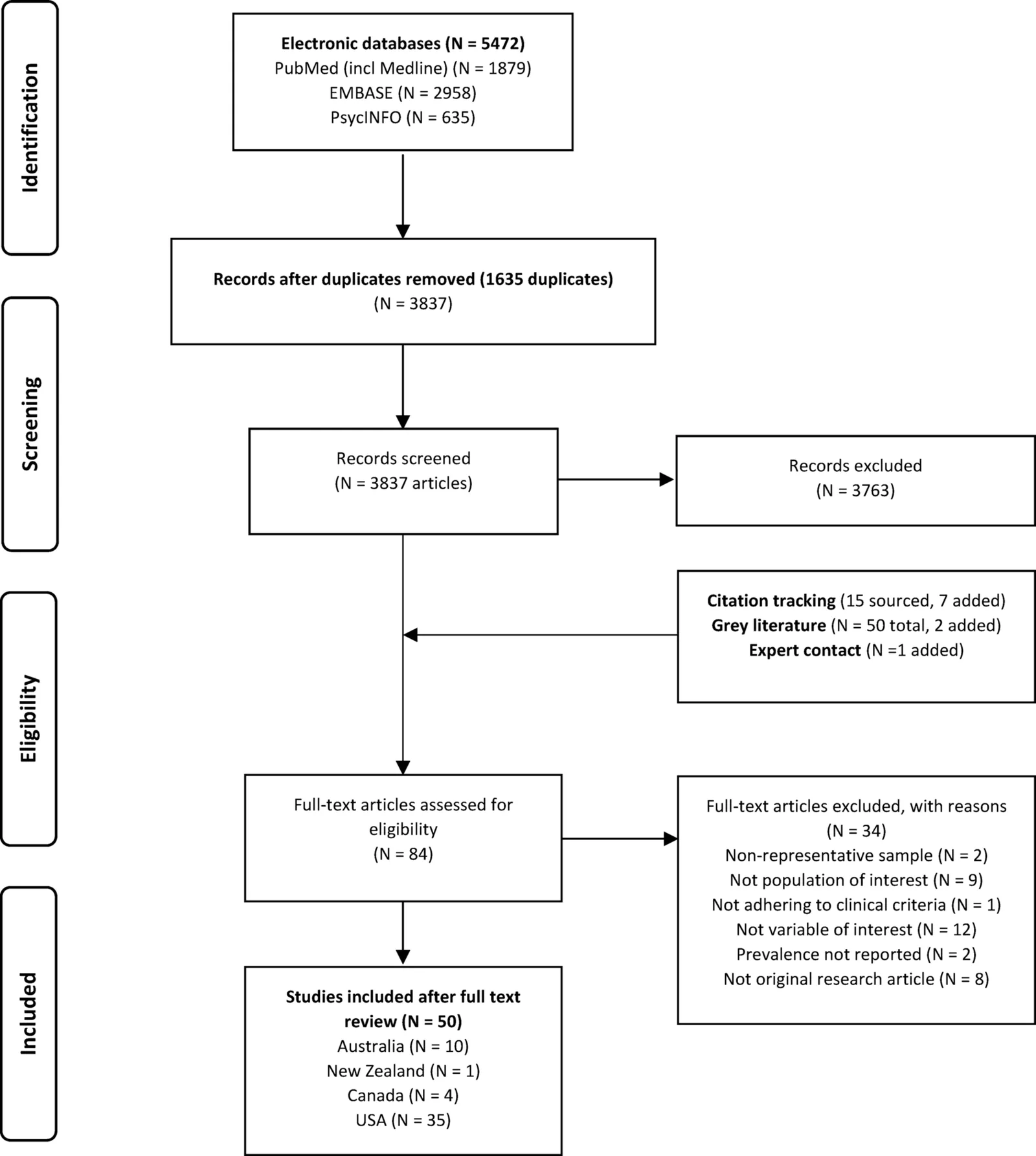
Study selection flowchart

A total of 50 studies on the prevalence of MSDs met eligibility criteria (see Fig. 1). This included ten studies from Australia, one from New Zealand, four from Canada and 35 from the USA. The number of representative prevalence surveys that these studies drew data from were nine from Australia, one from New Zealand, four from Canada and ten from the USA. See Table 1 for a summary of included studies and their characteristics.

Across all four countries, the most amount of data was available on mood disorders (major depressive disorder (MDD), major depressive episodes, dysthymia and bipolar disorder), anxiety disorders and substance use disorders (SUDs). The pooled estimates for lifetime MDD and post-traumatic stress disorder (PTSD) from the USA were drawn from four studies, the most data available across all four countries for any MSD. The least amount of data was available on agoraphobia, schizophrenia, obsessive-compulsive disorder and personality disorders, with one study reporting on each. Lifetime estimates were most measured (32 studies), followed by 12-month prevalence (23 studies) and point prevalence (15 studies).

Of the 50 identified studies, 13 reported on sociodemographic correlates of MSDs: four studies from Australia, one from New Zealand, three from Canada and five from the USA.

### Study quality appraisal

Study quality was assessed using the JBI Critical Appraisal Checklist for Prevalence Studies [22]. The majority of included studies were rated as high quality, likely reflecting the methodological rigour of large, population-based surveys, with two studies rated as low quality. Full item-level assessments are provided in the appendix (p.11).

### Variation in data coverage and collection

The type of surveys across the countries of interest varied greatly. They were either national level general population surveys or were regional surveys, comprised of smaller surveys of First Nations residents in selected areas (see Table 1). Australia’s studies were all regional, with no nationally representative data available. New Zealand had a national survey that oversampled First Nations peoples to achieve a representative number and produced comprehensive diagnostic data. Canadian had one national study, with the remaining three studies as regional; one produced a diagnostic prevalence estimate of MDD for females, and the remaining two produced a prevalence estimate of MDD using a symptom scale [27]. The USA had the most amount of data, with many studies using national datasets.

There was considerable variation in sampling and data collection strategies across studies (See Table 1). Most national surveys did not have a distinct sampling strategy to achieve a representative sample of First Nations peoples, whereas smaller, regional surveys tended to use targeted sampling of First Nations peoples to achieve a representative group. There were a range of diagnostic instruments used to produce prevalence estimates. The Composite International Diagnostic Interview 3.0 (CIDI 3.0) [28] was most commonly used in larger population surveys. Smaller regional surveys also used clinical interviews by trained professionals to produce diagnostic data. In addition to diagnostic data, there were nine surveys using symptom scales to approximate the prevalence of major depressive episodes, alcohol abuse, alcohol dependence, cannabis abuse and cannabis dependence (see Table 3).

**Table 3:** Prevalence estimates for MSDs using symptom scales across Australia, New Zealand, Canada and the United States of America

Disorder Mental Disorders	**Prevalence Type**	Australia			New Zealand			Canada			United States of America		
		Prevalence (%)	CI (95%)	Number of studies	Prevalence (%)	CI (95%)	Number of studies	Prevalence (%)	CI (95%)	Number of studies	Prevalence (%)	CI (95%)	Number of studies
Major Depressive Disorder	Point	18.1–45.5^*b*^	18.1–29.6 16.7–76.6	2	-	-	-	39.9–44.0 ^*b*^ (both) 44.0 (female) 35.5 (male)	36.3–41.6 39.3–48.7 (both) 40.6–47.3 (female) 30.4–39.4 (male)	2	14.1^*c*^ (both) 15.7^*c*^ (female) 8.9^*c*^ (male)	10.7–18.0 (both) 11.7–20.3 (female) 5.6–12.9 (male)	4 3 3
Any eating disorder	Point	27	19.1–37.0	1									
Substance Use Disorders													
Any substance use disorder	12-month	10.3	7.5–14.0	1	-	-	-	-	-	-	-	-	-
Substance Abuse	12-month	3.1	1.6–5.7	1	-	-	-	-	-	-	-	-	-
Substance Dependence	12-month	2.4	1.2–4.7	1	-	-	-	-	-	-	-	-	-
Alcohol Abuse	12-month	42.7	37.6–48.0	1	-	-	-	-	-	-	-	-	-
	Lifetime	73.8	67.4–79.4	1	-	-	-	-	-	-	-	-	-
Alcohol Dependence	12-month	6.3	4.2–9.4	1	-	-	-	-	-	-	-	-	-
	Lifetime	33.5	27.3–40.1	1	-	-	-	-	-	-	-	-	-
Cannabis Abuse	12-month	14.5	11.0–18.8	1	-	-	-	-	-	-	-	-	-
Cannabis Dependence	Point	34.3	25.8–42.5	1	-	-	-	-	-	-	-	-	-
	12-month	3.5	2.0–6.2	1	-	-	-	-	-	-	-	-	-
	Lifetime	5.9	3.1–9.8	1	-	-	-	-	-	-	-	-	-

Note: Only available estimates are provided. Single estimates (where only one estimate was available, ^b^Ranges (where there were two estimates reported), ^c^Pooled prevalence estimates (where there were three or more estimates reported)

### Prevalence estimates for MSDs in First Nations adults

Prevalence estimates (lifetime, 12-month and point-prevalence) by sex for MSDs using diagnostic data across each country of interest are reported in Table 2. Q-tests were mostly significant, and I^2^ analyses ranged from 20.53 to 1.81 across meta-analyses, indicating high levels of heterogeneity across studies. The distribution of data points within funnel plots suggest evidence of publication bias (see appendix p.20–55).

Prevalence estimates are presented as single estimates where only one estimate was available, ranges where there were two estimates reported, and pooled prevalence estimates where there were three or more estimates reported. Tables 2 and 3 provide further clarification between these sources of prevalence data.

### Lifetime prevalence

Overall, lifetime prevalence rates of any MSDs amongst First Nations adults ranged from 41.8% (95% confidence interval: 39.2–44.4%) in the USA to 66.9% (62.7–70.8%) in Australia. MDD ranged from 13.7% (8.2–20.2%) in the USA to 27.1% (24.3–29.9%) in Canada. Generalised anxiety disorder ranged from 4.5% (2.1–8.1%) in Australia to 20.8% (18.2–23.4%) in Canada. PTSD ranged 9.7% (8.2–11.4%) in New Zealand to 20.8% (17.4–24.4%) in Australia. SUDs ranged from 26.4% (16.9–37.1%) in the USA to 41.5% (37.7–45.8%) in Australia. Substance abuse ranged from 5.1% (3.9–6.3%) in the USA to 14.3% (12.6–16.1%) in New Zealand. Substance dependence ranged from 3.9% (3.0–5.1%) in the USA to 28.8% (26.0–31.6%) in Canada. Alcohol abuse ranged from 14.4% (8.2–2.2%) in the USA to 24.4 (22.3–26.7%) in New Zealand, and alcohol dependence from 10.1% (8.7–11.7%) in New Zealand to 22.9 (19.5–26.7%) in Australia.

### 12-Month prevalence

The 12-month prevalence rates of any MSDs amongst First Nations adults ranged from 20.9% (18.8–23.1%) in the USA and 51.7% (47.30–49.7%) in Australia. MDD ranged from 6.9% (5.8–8.1%) in New Zealand and 24.6% (20.4–29.3%) in Australia. Generalised anxiety disorder ranged from 1.6% (0.1–2.7%) in the USA to 8.1% (5.7–11.4%) in Australia. PTSD ranged from 4.5% (3.6–5.7%) in New Zealand to 19.9% (16.1–24.5%) in Australia. SUD ranged from 8.6% (7.1–10.4%) in New Zealand and 18.8% (15.6–22.2) in Australia.

The 12-month rates of substance abuse ranged from 2.3% (1.4–3.3%) in the USA to 3.7% (2.8–4.8%) in New Zealand, and substance dependence from 1.9% (1.3–2.8%) in New Zealand to 4.8% (3.1–6.9%) in Australia. Alcohol abuse ranged from 5.4% (3.8–7.2%) in the USA to 7.4% (5.3–9.8%) in Australia, and both the lowest and highest rates of alcohol dependence were reported by two different studies from Australia, ranging from 2.2% (1.2–3.4%) and 9.9% (7.5–12.7%).

### Point-prevalence

There were two point-prevalence estimates of any MSDs ranging from 18.3% (16.2–20.6%) in New Zealand to 45.4% (41.1–29.7%) in Australia. MDD ranged from 10.3% (7.8–13.1%) to 45.5% (16.7–76.6%) in Australia. PTSD also had two estimates, ranging from 5.7% (3.1–9.3%) in the USA to 13.8% (11.0–16.9%) in Australia. All other MSDs had either none or only 1 estimate reported across all countries of interest.

Prevalence estimates tended to be lowest in the USA and highest in Australia across all recall periods for most MSDs. Prevalence rates using symptom scales tended to be higher than diagnostic estimates, as described in Table 3. Some variation in prevalence by sex was observed. Estimates for mood disorders and anxiety disorders tended to be higher amongst females. Female lifetime estimates for any mood disorder ranged from 33.6% (28.9–38.5%) in the USA to 38.9% (33.6–44.3%) in Australia, whereas for males, they ranged from 22.5% (18.1–27.6%) in the USA to 31.9% (25.5–38.7%) in Australia. Female lifetime estimates for anxiety disorders ranged from 24.4% (19.4–29.9%) in the USA to 49% (43.5–54.4%) in Australia; in males, they ranged from 14.1% (10.2–18.5%) in the USA to 32.9% (26.5–39.7%) in Australia. SUDs tended to be higher amongst males, with male lifetime rates of any SUD ranging from 42.9 (37.7–48.4%) in the USA to 54.6% (47.5–61.5%) in Australia, relative to female lifetime rates of 14.9% (11.8–18.5%) in the USA to 33.5% (28.5–38.8%) in Australia. Prevalence estimates for any mental disorder were higher amongst males, with female lifetime estimates ranging from 35.7% (31.3–40.3%) in the USA to 63.2% (57.8–68.3%) in Australia, and male lifetime estimates ranging from 47.1% (42.2–52.0%) in the USA to 72.9% (66.3–78.8%) in Australia. Note not all differences across sex reached statistical significance. Estimates by sex across all included studies are reported in the appendix (p. 13–19).

### Sociodemographic correlates of MSDs in First Nations adults

The sociodemographic correlate data, taken from eleven studies, is summarised in Table 4. Only statistically significant correlates have been reported for the purposes of this summary. Being female was associated with higher prevalence relative to males in six studies, including for lifetime prevalence of any MSD and PTSD and point-prevalence of MDD. Being single/unmarried was associated with a higher point prevalence of MDD, and a higher lifetime prevalence of PTSD. Being unemployed and not completing high school were associated with a higher point-prevalence of MDD. Earning under half of the median national income and experiences of financial stress were each associated with higher lifetime prevalence of any MSD, and higher point prevalence of MDD. Unstable housing (sleeping rough or being homeless) was associated with higher 12-month prevalence of any MSD. Findings on age were mixed and varied by disorder, with younger ages associated with higher lifetime prevalence of any MSD and any SUD, and older ages associated with higher lifetime prevalence of PTSD and point-prevalence of MDD. Findings on regionality (urban vs. regional) were also mixed. Urban locations were a protective factor against the point-prevalence of MDD in two studies from the USA and Australia, while another Australian study found that being from a remote/very remote location was protective against any MSD, any mood disorder, any anxiety disorder and any substance disorder across the lifetime.

**Table 4:** Sociodemographic correlates of MSDs in First Nations adults

Country	References		MSD		Diagnostic or symptom scale		Recall period		Increased risk		Decreased risk		Statistic		Comparison Group		Comparison statistic		Significance
**Prevalence data**																			
New Zealand	Baxter et al., 2006 [12]		Any mental or substance use disorder		Diagnostic		Lifetime		Female				33.6%		Male		24.8%		< 0.05
									Younger ages 16–24 25–44 45–64				33.2% 32.9% 23.7%		65+		7.9%		< 0.05
									Under half of median income				40.9%		Half median to median Median to 1.5x median 1.5x median and over		26.9% 23.7% 19.7%		< 0.05
Australia	Nasir et al., 2018 [38]		Any mental or substance use disorder		Diagnostic		Point				Remote/very remote		7.5%		Major cities Inner Regional Outer Regional		42.8% 47.8% 36.9%		< 0.05
							12-mo				Remote/very remote		12.2%		Major cities Inner Regional Outer Regional		48.5% 53.3% 44.3%		< 0.05
							Lifetime				Remote/very remote		27.1%		Major cities Inner Regional Outer Regional		56.2% 68.5% 54.2%		< 0.05
			Any mood disorder		Diagnostic		Point				Remote/very remote		7.5%		Major cities Inner Regional Outer Regional		13.6% 18.2% 16.4%		< 0.05
							12-mo				Remote/very remote		12.2%		Major cities Inner Regional Outer Regional		14.8% 23.0% 17.8%		< 0.05
							Lifetime				Remote/very remote		22.1%		Major cities Inner Regional Outer Regional		24.1% 40.4% 23.4%		< 0.05
			Any anxiety disorder		Diagnostic		Point				Remote/very remote		5.3%		Major cities Inner Regional Outer Regional		24.7% 36.4% 22.4%		< 0.05
							12-mo				Remote/very remote		5.3%		Major cities Inner Regional Outer Regional		27.5% 39.4% 25.6%		< 0.05
							Lifetime				Remote/very remote		21.8%		Major cities Inner Regional Outer Regional		31.8% 47.4% 31.9%		< 0.05
			Any substance use disorder		Diagnostic		Point				Remote/very remote		2.2%		Major cities Inner Regional Outer Regional		19.2% 10.0% 14.5%		< 0.05
							12-mo				Remote/very remote		9.9%		Major cities Inner Regional Outer Regional		22.8% 14.5% 44.3%		< 0.05
							Lifetime				Remote/very remote		17.1%		Major cities Inner Regional Outer Regional		39.2% 39.1% 36.4%		< 0.05
Australia	Shen et al., 2018 [41]		Major depressive disorder		Symptom scale		Point		Regional location				22.4%		Urban		9.3%		< 0.1
Canada	Muckle et al., 2017 [40]		Major depressive disorder		Symptom scale		Point		Single				47.6%		Married/defacto Separated/divorced/widowed		33.3% 31.7%		< 0.05
									Lower education				42.1%		> Highschool		33.3%		< 0.05
									Below median income				45.3%		>$20,000		31.2%		< 0.5
									Unemployed				45.9%		Employed		35.8%		< 0.5
									Lived in small communities				45.6%		Large community		34.4%		< 0.5
United States of America	Curyto et al., 1998 [39]		Major depressive disorder		Symptom scale		Point		Older age (75+)				27.4%		55–75		15.9%		< 0.05
									Female				20.4%		Male		14.5%		< 0.05
									Not married				24.1		Married		13.1		< 0.05
									Urban				25.4%		Reservation Rural		17.5 8.5		< 0.05
United States of America	Dillard et al., 2012 [91]		Major depressive disorder		Symptom scale		Point		Female				20%		Male		13%		< 0.0001
									Lower education				22%		> Highschool/GED		16%		< 0.0001
									Unemployed				21%		Employed		15%		< 0.0001
									Below median income				21%		Above median income		12%		< 0.0001
United States of America	Sahota et al., 2008 [93]		Major depressive disorder		Symptom scale		Point		Female				12.8%		Male		7.5%		< 0.001
Odds ratios*																			
Australia	Basit et al., 2024 [3]		Any mental or substance use disorder		Diagnostic		Lifetime		Financial stress (unable to pay bills)				1.9 (1.1–3.2)		Able to pay bills		1.0		< 0.05
									Financial stress (unable to afford groceries)				2.0 (1.1–3.5)		Able to afford groceries		1.0		< 0.05
									Financial stress (seeking financial help from friends/family)				2.0 (1.3–2.3)		Did not seek financial help from friends/family		1.0		< 0.05
			Post-traumatic stress disorder						Single				1.7 (1.1–2.7)		Married/defacto		1.0		< 0.05
Australia	Ferrari et al., 2023 [45]		Any mental or substance use disorder		Diagnostic		12-mo		Unstable housing (sleeping rough/homeless/other)				6.0 (1.2–31.1)		Homeowner/renting/staying with friends/family		1.0		< 0.05
									Financial stress (unable to pay bills)				2.0 (1.2–3.3)		Able to pay bills		1.0		< 0.05
									Financial stress (unable to afford groceries)				2.0 (1.2–3.4)		Able to afford groceries		1.0		< 0.05
									Financial stress (sought assistance from welfare)				2.1 (1.3–3.3)		Did not seek assistance from welfare		1.0		< 0.05
									Financial stress (sought financial help from friend/family)				2.0 (1.3–3.2)		Did not seek financial help from friends/family		1.0		< 0.05
Canada	Froese et al., 2008 [59]		Major depressive disorder		Symptom scale		Point		Female				1.74 (0.7–2.8)		Male		1.0		< 0.05
United States of America	Beals et al., 2005a [31]		Any substance use disorder		Diagnostic		Lifetime		Younger age group (15–24)				1.5 (1.1–2.1)		> 45		1.0		< 0.05
United States of America	Beals et al., 2013 [64]		Post-traumatic stress disorder		Diagnostic		Lifetime		Female				2.16 (1.66–2.83)		Male				< 0.001
									Older ages 25-34 35-44 45+				1.90 (1.28–2.80) 1.91 (1.30–2.81) 2.41 (1.65–3.52)		15–24		1.0		< 0.001

* Results are presented as odds ratios where there was insufficient data available to estimate prevalence

## Discussion

High-quality data on prevalence and sociodemographic correlates of MSDs is important in assessing levels of mental health need and developing recommendations for mental health services. This systematic review summarised best-estimates of MSD prevalence and describe related sociodemographic correlates among First Nations adults across the four countries of interest, identifying similarities and trends where possible. Few representative prevalence surveys were identified, with 50 research papers reporting on prevalence and corresponding sociodemographic correlates data from a total of 24 unique population-level representative surveys across the countries of interest. Despite few identified studies and the heterogeneity introduced through differences in data collection methodology, a few general trends emerged.

### Prevalence

Lifetime, 12-month and point prevalence of MSDs across all First Nations populations were high, particularly when considering prevalence rates in their relative non-Indigenous populations [12, 29–31]. This follows trends reflected in measures of overall psychological distress amongst First Nations peoples relative to the general population [5–7] and underlines the need to understand the contributing factors resulting in this health disparity.

The patterns of prevalence within MSDs were similar to distributions of MSD prevalence in the general population for the identified countries, where anxiety and depressive disorders are most common [23]. Given the known cultural differences in notions of health and wellbeing [32] and the complex interplay of individual and environmental risk factors influencing the occurrence of MSDs, more research is needed to quantify the relative contribution of these risk factors across First Nations and non-Indigenous populations to better interpret and anticipate these similarities and differences.

When comparing common MSDs across countries of interest, prevalence rates were typically highest for Australia, sometimes Canada, and lowest in the USA, persisting across recall period and sex. We urge caution in interpreting these differences due to the heterogeneity across studies. Australia and Canada had relatively few identified studies and smaller sample sizes relative to the greater number of large cohort studies in the USA, which makes it difficult to determine how much of this trend is reflective of true differences in prevalence (as opposed to measurement error or missing data). That said, MSD prevalence amongst First Nations Australians was also substantially higher than First Nations New Zealanders, which was surprising given the countries’ proximity and socio-economic similarities in healthcare structures and social welfare systems, relative to the USA where healthcare and welfare systems are markedly different [33]. Trends in health data such as life expectancy and mortality statistics also suggests that the health disparities First Nations Australians face relative to the non-Indigenous population are greater than the disparities faced by First Nations peoples in other developed countries [34]. Like other countries impacted by colonisation, institutional, interpersonal, and internalised racism is entrenched in Australian society and systemically disadvantages First Nations Australians [34]. More work needs to be done to identify the drivers of higher prevalence of MSDs in First Nations peoples of Australia and identify helpful strategies in mental health service responses in countries where estimates are comparatively lower.

Prevalence tended to be higher amongst females for mood and anxiety disorders and higher amongst males for SUDs, which follows trends observed in MSD prevalence globally [35]. Societal gender inequalities are known to impact on increased prevalence of several MSDs in women regardless of ethnicity, through impacts on social and economic outcomes and interpersonal experiences of violence, childhood sexual abuse, and discrimination [36]. Observed sex differences may also be influenced by mental health stigma, gendered norms around emotional expression, and differential help-seeking and diagnostic practices, which may contribute to underreporting or underdiagnosis of mood and anxiety disorders among men [37]. Given the additional cultural and historic factors unique to the experience of First Nations peoples, the compounded impact on First Nations women who experience mood and anxiety disorders, and First Nations men who experience SUDs must be given due consideration [4]. The intersectionality between gender and Indigenous status is of particular importance when planning targeted health services and initiatives. Again, the importance of accurate population level data in First Nations peoples becomes paramount in understanding and responding to mental health needs in a nuanced way.

### Correlates

Few trends in the sociodemographic correlates of MSDs could be meaningfully extracted. The most consistent correlate appeared to be sex, where being female was associated with a higher risk of several MSDs, as discussed above. One Australian study found higher rate of MSD in males than females [38], but potential explanations were not explored by authors. Socioeconomic factors, such as having a lower income, experiencing financial stress, unstable housing, lower education and unemployment were also risk factors for some MSDs. Additionally, some studies reported younger ages were at increased risk of having any MSD or SUD over the lifetime and older ages at higher risk for PTSD over the lifetime and point-prevalence of MDD. As these findings were from disparate studies, and given the heterogeneity in study design, meaningful interpretations on overall trends or patterns are not possible. Similarly, findings on regionality were also mixed. Multiple factors may contribute to how urbanicity may be both protective and a risk factor and explain some of the variations in identified findings. Some studies theorised that lower rates of MSD in Reserve and remote residents may be a result of those residents having a preserved connection to traditional lands and community [38–40]. Others that found higher rates of MSD in rural and remote residents suggested that less access to healthcare services, resource allocation and greater social inequity may contribute to poorer mental health outcomes [41]. Few studies in this review examined protective factors against MSDs other than regionality. This likely reflects the limited scope of diagnostic epidemiological surveys, which seldom include culturally grounded measures of connection to culture, community, land, or identity.

### Limitations and recommendations

The findings of this review are limited to the Indigenous populations and countries represented in the included studies and may not be generalisable to other colonised Indigenous First Nations populations. Differences in colonial histories, cultural contexts, and health systems may influence both the prevalence and expression of mental and substance use disorders. Extending interpretation beyond these contexts would require a broader evidentiary base and theoretical framework, which was beyond the scope of this review.

The small number of studies meeting inclusion criteria presented several limitations for data analysis and interpretation. Publication bias may have meant that not all studies meeting inclusion criteria were identified. Due to few datapoints per MSD, meta-analyses could not be conducted for all disorders, recall period, location, and sex disaggregation. We attempted to maximise use of available data by presenting single or ranged estimates where available for prevalence however these may not represent representative summary metrics for the target population.

There are several overarching reasons that may account for the small number of epidemiological surveys in this systematic review. Due to population sizes and distribution, First Nations people are often underrepresented in national epidemiological surveys as the sampling methodology cannot identify sufficient participants to derive estimates for minority populations [42–44]. Representative samples can be established by oversampling from First Nations populations, as was done in the New Zealand Mental Health Survey [12], but this strategy is not often employed given the additional resourcing required. Mixed-methods sampling strategies with randomised household sampling and convenience sampling, utilised in one Australian study, can serve as an alternative, but may not identify a fully representative sample [45, 46]. These structural challenges are particularly evident in the Canadian context, where few population-level surveys have incorporated fully standardised diagnostic assessments while also achieving representative coverage of First Nations populations, including those living on-reserve. In addition, the strong emphasis on Indigenous data governance and sovereignty in Canada [47], while essential for ethical research practice, may limit the secondary use and publication of epidemiological data in ways that reduce their visibility in peer-reviewed syntheses. Contextually, past research on MSDs in First Nations groups has often perpetuated and entrenched the process of colonisation, objectifying and disempowering communities [48, 49]. This has a direct effect on study participation rates, and researchers must embed decolonising research practices into survey methods to improve acceptability and reduce harm [50].

The heterogeneity of survey sampling strategies, study designs, participant characteristics and diagnostic assessments, combined with sparse and unevenly distributed nature of the available data, restricted much of the synthesis to descriptive comparisons, and summary estimates should be interpreted cautiously. As more data become available from diagnostic mental health surveys of First Nations populations, future analyses will be able to incorporate more statistically robust subgroup and meta-regression approaches. In addition, although the review aimed to summarise both prevalence and sociodemographic correlates of MSDs, the search strategy prioritised studies reporting on prevalence outcomes, which may have reduced sensitivity for identifying correlate-focused surveys. However, the central finding of this review is the limited availability and uneven coverage of high-quality, comparable data, and pooled estimates are therefore best interpreted as indicative summaries rather than precise or stable prevalence values.

The lengths to which studies went to validate survey instruments for use with First Nation peoples and make survey administration culturally appropriate was not always clear. While structured diagnostic instruments are considered to be more accurate and robust than non-specific indicators of psychological distress or symptom scales [38], they draw from the DSM [17] or ICD [18]. These classifications systems utilise Western “deficit” notions of health, where health is conceptualised as an absence of illness. This is different to First Nations conceptions of wellbeing; where wellbeing consists of physical, emotional, and spiritual elements, and extends beyond the individual to include ancestors, family and community, land and nature [4, 48, 51]. The assessment and treatment of illness is much broader than can be captured by Western frameworks [32]. Until specialised diagnostic instruments designed specifically for use with First Nations peoples are established, adaptations to data-collection processes to make structured diagnostic assessments more culturally suitable may be a way forward [52]. An example of embedding cultural adaptations to successfully deliver an epidemiological survey in an urban population in Australia has been recently published [45, 46].

## Conclusion

This systematic review highlights the need for comprehensive, representative data on MSDs and their corresponding sociodemographic correlates in First Nations peoples. The data to date is piecemeal and heterogeneous across sampling strategies, study designs and assessments. Prioritising the funding and administration of nationally representative epidemiological surveys for First Nations peoples, including for child and adolescent populations [53] is critical for the allocation of targeted services that can affect meaningful change. Considerations must be taken around survey methods and assessments to ensure they are culturally responsive to community needs. Until more research is conducted, summarised metrics on prevalence and social determinants of MSDs across First Nations peoples in Australia, New Zealand, Canada and the USA may highlight commonalities stemming from their shared economic, cultural and historic circumstances. In turn, learnings from public health initiatives in one country hold promise for application in another.

## Electronic Supplementary Material

Below is the link to the electronic supplementary material.

Supplementary Material 1

## Data Availability

Data supporting the findings of this systematic review are available from the authors upon request.
