# Supplementary material for: A systematic review of the prevalence and correlates of mental and substance use disorders in first nations peoples in Australia, New Zealand, Canada and the United States of America
Source: Soc Psychiatry Psychiatr Epidemiol. 2026 Apr 29;61(9):1661–85. doi: 10.1007/s00127-026-03104-5 (PMC13578000; doi:10.1007/s00127-026-03104-5)
Supplement: Supplementary file 1 [file 127_2026_3104_MOESM1_ESM.docx]

Appendix

[PRISMA Checklist	1](#_Toc224899754)

[Search strings and methodology	3](#_Toc224899755)

[Search string	3](#_Toc224899756)

[Inclusion/Exclusion criteria	5](#_Toc224899757)

[Data extraction	6](#_Toc224899758)

[Included disorders in extracted data	6](#_Toc224899759)

[Data and analysis	8](#_Toc224899760)

[Disorders included in analysis by study	8](#_Toc224899761)

[Quality appraisal of included studies (JBI checklist)	11](#_Toc224899762)

[Gender specific prevalence estimates for MSD’s using diagnostic data across Australia, New Zealand, Canada and the United States of America	13](#_Toc224899763)

[Forest plots and funnel plots for meta-analyses	20](#_Toc224899764)

[Alcohol abuse, 12-month, both	20](#_Toc224899765)

[Alcohol abuse, 12-month, female	20](#_Toc224899766)

[Alcohol abuse, 12-month, male	21](#_Toc224899767)

[Alcohol abuse, lifetime, both	22](#_Toc224899768)

[Alcohol abuse, lifetime, female	22](#_Toc224899769)

[Alcohol abuse, lifetime, male	23](#_Toc224899770)

[Alcohol dependence, 12-month, both	24](#_Toc224899771)

[Alcohol dependence, 12-month, female	24](#_Toc224899772)

[Alcohol dependence, 12-month, male	25](#_Toc224899773)

[Alcohol dependence, lifetime, both	26](#_Toc224899774)

[Alcohol dependence, lifetime, female	26](#_Toc224899775)

[Alcohol dependence, lifetime, male	27](#_Toc224899776)

[Any anxiety disorder, 12-month, both	28](#_Toc224899777)

[Any anxiety disorder, 12-month, female	28](#_Toc224899778)

[Any anxiety disorder, 12-month, male	29](#_Toc224899779)

[Any anxiety disorder, lifetime, both	30](#_Toc224899780)

[Any anxiety disorder, lifetime, female	30](#_Toc224899781)

[Any anxiety disorder, lifetime, male	31](#_Toc224899782)

[Any substance use disorder, 12-month, both	32](#_Toc224899783)

[Any substance use disorder, lifetime, both	32](#_Toc224899784)

[Dysthymia, 12-month, both	33](#_Toc224899785)

[Dysthymia, 12-month, female	34](#_Toc224899786)

[Dysthymia, 12-month, male	34](#_Toc224899787)

[Dysthymia, lifetime, both	35](#_Toc224899788)

[Dysthymia, lifetime, female	36](#_Toc224899789)

[Dysthymia, lifetime, male	36](#_Toc224899790)

[Generalised anxiety disorder, 12-month, both	37](#_Toc224899791)

[Generalised anxiety disorder, 12-month, male	38](#_Toc224899792)

[Generalised anxiety disorder, 12-month, female	38](#_Toc224899793)

[Generalised anxiety disorder, lifetime, both	39](#_Toc224899794)

[Generalised anxiety disorder, lifetime, male	40](#_Toc224899795)

[Generalised anxiety disorder, lifetime, female	40](#_Toc224899796)

[Major depressive disorder, 12-month, both	41](#_Toc224899797)

[Major depressive disorder, 12-month, female	42](#_Toc224899798)

[Major depressive disorder, 12-month, male	42](#_Toc224899799)

[Major depressive disorder, lifetime, both	43](#_Toc224899800)

[Major depressive disorder, lifetime, female	44](#_Toc224899801)

[Major depressive disorder, lifetime, male	44](#_Toc224899802)

[Panic disorder, 12-month, both	45](#_Toc224899803)

[Panic disorder, 12-month, female	46](#_Toc224899804)

[Panic disorder, 12-month, male	46](#_Toc224899805)

[Panic disorder, lifetime, both	47](#_Toc224899806)

[Panic disorder, lifetime, female	48](#_Toc224899807)

[Panic disorder, lifetime, male	48](#_Toc224899808)

[Post-traumatic stress disorder, 12-month, both	49](#_Toc224899809)

[Post-traumatic stress disorder, lifetime, both	50](#_Toc224899810)

[Post-traumatic stress disorder, lifetime, male	50](#_Toc224899811)

[Post-traumatic stress disorder, lifetime, female	51](#_Toc224899812)

[Substance abuse, 12-month, both	52](#_Toc224899813)

[Substance dependence, 12-month, both	52](#_Toc224899814)

[Prescription opioid use disorder, lifetime, both	53](#_Toc224899815)

[Major depressive disorder (symptom scale), point-prevalence, both	54](#_Toc224899816)

[Major depressive disorder (symptom scale), point-prevalence, female	54](#_Toc224899817)

[Major depressive disorder (symptom scale), point-prevalence, male	55](#_Toc224899818)

## PRISMA Checklist

| **Section and Topic** | **Item #** | **Checklist item** | **Location where item is reported** |
| --- | --- | --- | --- |
| **TITLE** | | |  |
| Title | 1 | Identify the report as a systematic review. | 1 |
| **ABSTRACT** | | |  |
| Abstract | 2 | See the PRISMA 2020 for Abstracts checklist. | 2 |
| **INTRODUCTION** | | |  |
| Rationale | 3 | Describe the rationale for the review in the context of existing knowledge. | 3 |
| Objectives | 4 | Provide an explicit statement of the objective(s) or question(s) the review addresses. | 3 |
| **METHODS** | | |  |
| Eligibility criteria | 5 | Specify the inclusion and exclusion criteria for the review and how studies were grouped for the syntheses. | 3-4 |
| Information sources | 6 | Specify all databases, registers, websites, organisations, reference lists and other sources searched or consulted to identify studies. Specify the date when each source was last searched or consulted. | 3 |
| Search strategy | 7 | Present the full search strategies for all databases, registers and websites, including any filters and limits used. | 3, appendix 3-5 |
| Selection process | 8 | Specify the methods used to decide whether a study met the inclusion criteria of the review, including how many reviewers screened each record and each report retrieved, whether they worked independently, and if applicable, details of automation tools used in the process. | 3, appendix 5-6 |
| Data collection process | 9 | Specify the methods used to collect data from reports, including how many reviewers collected data from each report, whether they worked independently, any processes for obtaining or confirming data from study investigators, and if applicable, details of automation tools used in the process. | 3 |
| Data items | 10a | List and define all outcomes for which data were sought. Specify whether all results that were compatible with each outcome domain in each study were sought (e.g. for all measures, time points, analyses), and if not, the methods used to decide which results to collect. | 3 |
|  | 10b | List and define all other variables for which data were sought (e.g. participant and intervention characteristics, funding sources). Describe any assumptions made about any missing or unclear information. | 3-5, appendix 5-6 |
| Study risk of bias assessment | 11 | Specify the methods used to assess risk of bias in the included studies, including details of the tool(s) used, how many reviewers assessed each study and whether they worked independently, and if applicable, details of automation tools used in the process. | 3-4 |
| Effect measures | 12 | Specify for each outcome the effect measure(s) (e.g. risk ratio, mean difference) used in the synthesis or presentation of results. | 4-5 |
| Synthesis methods | 13a | Describe the processes used to decide which studies were eligible for each synthesis (e.g. tabulating the study intervention characteristics and comparing against the planned groups for each synthesis (item #5)). | 3-5, appendix 5-6 |
|  | 13b | Describe any methods required to prepare the data for presentation or synthesis, such as handling of missing summary statistics, or data conversions. | 4-5, appendix 6 |
|  | 13c | Describe any methods used to tabulate or visually display results of individual studies and syntheses. | 4-5 |
|  | 13d | Describe any methods used to synthesize results and provide a rationale for the choice(s). If meta-analysis was performed, describe the model(s), method(s) to identify the presence and extent of statistical heterogeneity, and software package(s) used. | 4-5 |
|  | 13e | Describe any methods used to explore possible causes of heterogeneity among study results (e.g. subgroup analysis, meta-regression). | 5 |
|  | 13f | Describe any sensitivity analyses conducted to assess robustness of the synthesized results. | 5 |
| Reporting bias assessment | 14 | Describe any methods used to assess risk of bias due to missing results in a synthesis (arising from reporting biases). | 5 |
| Certainty assessment | 15 | Describe any methods used to assess certainty (or confidence) in the body of evidence for an outcome. | 5, appendix 11-55 |
| **RESULTS** | | |  |
| Study selection | 16a | Describe the results of the search and selection process, from the number of records identified in the search to the number of studies included in the review, ideally using a flow diagram. | 5-6 |
|  | 16b | Cite studies that might appear to meet the inclusion criteria, but which were excluded, and explain why they were excluded. | Appendix 6 |
| Study characteristics | 17 | Cite each included study and present its characteristics. | 22-29 |
| Risk of bias in studies | 18 | Present assessments of risk of bias for each included study. | Appendix 20-55 |
| Results of individual studies | 19 | For all outcomes, present, for each study: (a) summary statistics for each group (where appropriate) and (b) an effect estimate and its precision (e.g. confidence/credible interval), ideally using structured tables or plots. | 30-39 |
| Results of syntheses | 20a | For each synthesis, briefly summarise the characteristics and risk of bias among contributing studies. | 7 |
|  | 20b | Present results of all statistical syntheses conducted. If meta-analysis was done, present for each the summary estimate and its precision (e.g. confidence/credible interval) and measures of statistical heterogeneity. If comparing groups, describe the direction of the effect. | 7-8, appendix 20-55 |
|  | 20c | Present results of all investigations of possible causes of heterogeneity among study results. | Appendix 20-55 |
|  | 20d | Present results of all sensitivity analyses conducted to assess the robustness of the synthesized results. | Appendix 20-55 |
| Reporting biases | 21 | Present assessments of risk of bias due to missing results (arising from reporting biases) for each synthesis assessed. | 4, 7 |
| Certainty of evidence | 22 | Present assessments of certainty (or confidence) in the body of evidence for each outcome assessed. | 7-8 |
| **DISCUSSION** | | |  |
| Discussion | 23a | Provide a general interpretation of the results in the context of other evidence. | 9-12 |
|  | 23b | Discuss any limitations of the evidence included in the review. | 11-12 |
|  | 23c | Discuss any limitations of the review processes used. | 11-12 |
|  | 23d | Discuss implications of the results for practice, policy, and future research. | 12 |
| **OTHER INFORMATION** | | |  |
| Registration and protocol | 24a | Provide registration information for the review, including register name and registration number, or state that the review was not registered. | 4 |
|  | 24b | Indicate where the review protocol can be accessed, or state that a protocol was not prepared. | 4 |
|  | 24c | Describe and explain any amendments to information provided at registration or in the protocol. | 4 |
| Support | 25 | Describe sources of financial or non-financial support for the review, and the role of the funders or sponsors in the review. | 14 |
| Competing interests | 26 | Declare any competing interests of review authors. | 14 |
| Availability of data, code and other materials | 27 | Report which of the following are publicly available and where they can be found: template data collection forms; data extracted from included studies; data used for all analyses; analytic code; any other materials used in the review. | 14 |

*From:* Page MJ, McKenzie JE, Bossuyt PM, Boutron I, Hoffmann TC, Mulrow CD, et al. The PRISMA 2020 statement: an updated guideline for reporting systematic reviews. BMJ 2021;372:n71. doi: 10.1136/bmj.n71. For more information, visit: <http://www.prisma-statement.org/>

## Search strings and methodology

### Search string

(((((((("surveys and questionnaires"[MeSH Terms])

OR ("health surveys"[MeSH Terms]))

OR ("interview, psychological"[MeSH Terms]))

OR (Survey[Title/Abstract]))

OR (Survey*[Title/Abstract])

OR (Questionnaire*[Title/Abstract])

OR (Interview*[Title/Abstract])

AND

((((("prevalence"[MeSH Terms])

OR ("epidemiology"[MeSH Terms]))

OR ("cross sectional studies"[MeSH Terms]))

OR (prevalence[Title/Abstract]))

OR (epidemiolog*[Title/Abstract])

AND

(((((((((("indigenous canadians"[MeSH Terms])

OR ("oceanic ancestry group"[MeSH Terms]))

OR ("indigenous peoples"[MeSH Terms]))

OR ("american native continental ancestry group"[MeSH Terms]))

OR (Aborigin*[Title/Abstract]))

OR (Indigenous*[Title/Abstract]))

OR ("First Nation*"[Title/Abstract]))

OR ("Torres Strait"[Title/Abstract]))

OR ("First Australian*"[Title/Abstract]))

OR ("American Indian*"[Title/Abstract])

OR ("Native American*"[Title/Abstract])

OR ("North American Native*"[Title/Abstract])

OR ("Pacific Northwest American*"[Title/Abstract])

OR ("Pacific Northwest Indian*"[Title/Abstract])

OR (Amerindian*[Title/Abstract])

OR (Inuit[Title/Abstract])

OR ("Alaskan Native*"[Title/Abstract])

OR (Hawaiian[Title/Abstract])

OR (Eskimo*[Title/Abstract])

OR (Maori[Title/Abstract])

OR (Metis[Title/Abstract])

AND

((((((((((((((((((((((((((((((((((((((("mental disorders"[MeSH Terms])

OR ("substance related disorders"[MeSH Terms]))

OR ("psychological disorder*"[Title/Abstract]))

OR ("psychiatric disorder*"[Title/Abstract]))

OR ("mental disorder*"[Title/Abstract]))

OR ("mental illness*"[Title/Abstract]))

OR ("mental health"[Title/Abstract]))

OR ("mental and substance use disorder*"[Title/Abstract]))

OR ("substance use disorder*"[Title/Abstract]))

OR ("substance abuse"[Title/Abstract]))

OR ("substance related disorder*"[Title/Abstract]))

OR ("social and emotional wellbeing" [Title/Abstract]))

OR (Alcoholism[Title/Abstract]))

OR ("alcohol-related disorder*"[Title/Abstract]))

OR (addiction[Title/Abstract]))

OR (dependenc*[Title/Abstract]))

OR (depress*[Title/Abstract]))

OR (dysthymi*[Title/Abstract]))

OR (bipolar[Title/Abstract]))

OR (manic[Title/Abstract]))

OR (mania[Title/Abstract]))

OR ("anxiety"[Title/Abstract]))

OR ("generalised anxiety disorder*"[Title/Abstract]))

OR ("generalized anxiety disorder*"[Title/Abstract]))

OR ("anxiety disorder*"[Title/Abstract]))

OR ("post traumatic stress disorder*"[Title/Abstract]))

OR ("posttraumatic stress disorder*"[Title/Abstract]))

OR ("traumatic stress disorder*"[Title/Abstract]))

OR (trauma[Title/Abstract]))

OR (schizo*[Title/Abstract]))

OR (psychosis[Title/Abstract]))

OR (psychotic[Title/Abstract]))

OR (anorexia*[Title/Abstract]))

OR (bulimia*[Title/Abstract]))

OR ("eating disorder*"[Title/Abstract]))

OR (opioid*[Title/Abstract]))

OR (heroin[Title/Abstract]))

OR (cocaine[Title/Abstract]))

OR (marijuana[Title/Abstract])

OR (cannabis[Title/Abstract])

OR (amphetamin*[Title/Abstract])

OR (methamphetam*[Title/Abstract])

OR (((((drug*[Title/Abstract]

OR substance*[Title/Abstract]

OR alcohol [Title/Abstract])

AND

(abus*[Title/Abstract]))

OR (dependen*[Title/Abstract]))

OR (misus*[Title/Abstract]))

OR (addict*[Title/Abstract]))

### Inclusion/Exclusion criteria

Studies were **included** if they met all of the following criteria:

1. Publication date: Published from 1980 onwards. This aligns with publication of the DSM-III, which introduced operationalised diagnostic criteria underpinning modern psychiatric epidemiology. This publication date ensures currency of prevalence and correlate estimates and improve comparability of diagnostic definitions across studies.
2. Study design and focus: Epidemiological surveys identifying prevalent cases of mental and/or substance use disorders in adult populations, including associated sociodemographic correlates. Surveys reporting prevalence across any recall period were eligible.
3. Diagnostic framework: Surveys broadly adhering to diagnostic definitions as specified in the *Diagnostic and Statistical Manual of Mental Disorders* (DSM) or the *International Classification of Diseases and Related Health Problems* (ICD). This included:
   - Surveys using validated diagnostic instruments fully aligned with DSM or ICD criteria; and
   - Symptom-based measures that partially adhered to DSM or ICD criteria, provided they estimated mental and substance use disorders.
4. Population and representativeness: Studies using sampling strategies designed to produce estimates representative of the Indigenous population of the relevant geographic region, including population surveys reporting Indigenous-specific estimates and studies focused exclusively on Indigenous peoples.
5. Sufficient information on study design, sampling, and participant characteristics to permit assessment of data quality.
6. Primary empirical studies.
7. No restrictions on language of publication.

Studies were **excluded** if they met any of the following criteria:

1. Focused solely on non-specific psychological distress, general emotional health, or wellbeing, rather than mental and substance use disorders.
2. Used non-representative or high-risk subgroups where estimates would not reflect the broader Indigenous population (e.g., incarcerated populations, clinical samples, or other systematically biased samples).
3. Were secondary sources, including literature reviews, systematic reviews, meta-analyses, commentaries, or policy reports.
4. Lacked sufficient methodological detail to enable assessment of study quality.

### Data extraction

- The following information was extracted from all surveys meeting inclusion criteria: study citation and country, survey site, ethnic group, urbanicity, sampling strategy, year start and end of data collection, response rate, sample age, sample size, disorders assessed, survey instrument(s), recall period, and survey outputs of interest, (i.e. prevalence estimates and measures of correlation).
- Available lifetime, past year, and point prevalence estimates were extracted for all included MSDs, with measures of uncertainty where available.
- Due to the low number of included studies and the heterogeneity of their methodologies, several decisions were made when coding and extracting the data to maximise data use. Data from broad categories such as ‘mood and substance use disorders’ were not used as these groupings were rarely used across studies and were less informative than specific disorders. Where multiple records reported estimates from the same survey, the most informative record reporting on the most granular estimates was used, or the studies that used the diagnostic criteria that were most comparable to the diagnostic criteria used for the other studies reporting on that disorder were extracted. Where some studies reported on prevalence of bipolar I disorder and bipolar II disorder, those disorders were combined into an overall prevalence of bipolar disorder to increase comparability to other studies reporting on bipolar disorder. Similarly, for studies reporting on male and female estimates, those estimates were combined into an estimate for both genders to maximise opportunities for analysis.
- Examples of exclusions: one study reported cumulative prevalence, however the first wave consisted of adolescents aged 10-12 years, so was removed (Walls, 2020). One particular study, Braveheart, was removed from some analyses as it included nicotine dependence in its prevalence estimate of any mental disorder and any substance use disorder, where others did not. This produced an inflated estimate of those disorders and could not be included in those analyses. The same study also included participants up to age 99, where many other studies in the same meta-analysis only included participants up to age 54. As a result, the prevalence for the Braveheart study was sometimes higher than others. In these instances, it was retained.
- An estimate of uncertainty (either a standard error or 95% uncertainty interval) around the risk estimate will be extracted if reported, or calculated using the formula:

$SE= \sqrt{2.1\left( \frac{P\left( 1-P \right)}{N} \right)}$

Where N=sample size, P= proportion/quantity of interest

### Included disorders in extracted data

Mental Disorders: Any mental disorder, any mood disorder, any depressive disorder, major depressive disorder, dysthymia, bipolar disorder, mania, any anxiety disorder, generalised anxiety disorder, panic disorder, post-traumatic stress disorder, social anxiety disorder, agoraphobia, specific phobia, schizophrenia, obsessive-compulsive disorder, any eating disorder, anorexia, bulimia, attention deficit hyperactivity disorder.

Substance Use Disorders: Any substance use disorder, substance abuse, substance dependence, any alcohol use disorder, alcohol abuse, alcohol dependence, any cannabis use disorder, cannabis abuse, cannabis dependence, opioid use disorder, nicotine dependence.

Personality Disorders: Any personality disorder, avoidant personality disorder, dependent personality disorder, histrionic personality disorder, obsessive-compulsive personality disorder, paranoid personality disorder, schizoid personality disorder.

## Data and analysis

### Disorders included in analysis by study

| Study | Disorders included in analysis |
| --- | --- |
| Almeida et. Al., 2014 | Any depressive disorder |
| Basit et Al, 2024 | Major depressive disorder, generalised anxiety disorder, post-traumatic stress disorder |
| Baxter et. Al., 2006 | Any anxiety disorder, any mood disorder, major depressive disorder, bipolar disorder, any substance use disorder, eating disorder, any mental disorder, panic disorder, agoraphobia, specific phobia, social anxiety disorder, generalised anxiety disorder, post-traumatic stress disorder, obsessive-compulsive disorder, dysthymia, alcohol abuse, alcohol dependence, substance abuse, substance dependence, cannabis abuse, cannabis dependence, any alcohol use disorder, any substance use disorder, anorexia, bulimia |
| Beals et. Al., 2005a | Alcohol abuse, alcohol dependence, any anxiety disorder, any depressive disorder, any mental disorder, any substance use disorder, dysthymia, generalised anxiety disorder, major depressive disorder, panic disorder, post-traumatic stress disorder, substance abuse, substance dependence |
| Beals et. Al., 2005b | Post-traumatic stress disorder |
| Beals et. Al., 2013 | Post-traumatic stress disorder (correlates) |
| Blanco et Al., 2017 | Bipolar disorder |
| Bohanna et. Al., 2012 | Cannabis dependence |
| Brave Heart et. Al., 2016 | Alcohol abuse, alcohol dependence, antisocial personality disorder, any alcohol use disorder, any anxiety disorder, any mental disorder, any mood disorder, any substance use disorder, any personality disorder, avoidant personality disorder, bipolar disorder, dependent personality disorder, dysthymia, generalised anxiety disorder, histrionic personality disorder, major depressive disorder, nicotine dependence, obsessive-compulsive personality disorder, panic disorder, paranoid personality disorder, schizoid personality disorder, substance abuse, substance dependence, social anxiety disorder, specific phobia |
| Burt et. Al., 2020 | Eating disorder |
| Curyto et. Al., 1998 | Major depressive disorder |
| Dashputre et. Al., 2023 | Major depressive disorder |
| Dillard et. Al., 2012 | Major depressive disorder |
| Emerson et. Al., 2017 | Generalised anxiety disorder, major depressive disorder, post-traumatic stress disorder |
| Ferrari et. Al., 2023 | Any mental disorder, any substance use disorder, alcohol abuse, alcohol dependence, cannabis abuse, cannabis dependence, generalised anxiety disorder, major depressive disorder, substance abuse, substance dependence, post-traumatic stress disorder |
| Froese et. Al, 2008 | Major depressive disorder |
| Fuller-Thomson et. Al., 2020 | Bipolar disorder, major depressive disorder, generalised anxiety disorder, any alcohol use disorder, cannabis dependence, substance dependence |
| Goldstein et. Al., 2016 | Post-traumatic stress disorder |
| Grant et. Al., 2004 | Antisocial personality disorder, avoidant personality disorder, dependent personality disorder, histrionic personality disorder, obsessive-compulsive personality disorder, paranoid personality disorder, schizoid personality disorder |
| Grant et. Al., 2005a | Bipolar disorder |
| Grant et. Al., 2005b | Social anxiety disorder |
| Grant et. Al., 2006 | Panic disorder |
| Grant et. Al., 2008 | Borderline personality disorder |
| Grant et. Al., 2015 | Any alcohol use disorder |
| Grant et. Al., 2016 | Any substance use disorder |
| Hasin et. Al., 2005 | Major depressive disorder |
| Hasin et. Al., 2007 | Any alcohol use disorder, alcohol abuse, alcohol dependence |
| Hasin et. Al., 2016 | Any substance use disorder |
| Hasin et. Al., 2018 | Major depressive disorder |
| Huang et. Al., 2006 | Prescription opioid use disorder |
| Kerridge et. Al., 2015 | Prescription opioid use disorder |
| Kerridge et. Al., 2018 | Any cannabis use disorder |
| Kinzie et. Al., 1992 | Alcohol abuse, alcohol dependence, any alcohol use disorder, substance abuse, major depressive disorder, generalised anxiety disorder, panic disorder, agoraphobia, specific phobia, post-traumatic stress disorder, bipolar disorder. |
| Koss et. Al., 2003 | Alcohol abuse, alcohol dependence |
| MacMillan et. Al., 2008 | Major depressive disorder |
| Muckle et. Al., 2017 | Major depressive disorder |
| Nadew et. Al., 2012 | Alcohol abuse, alcohol dependence, agoraphobia, any anxiety disorder, cannabis abuse, cannabis dependence, dysthymia, generalised anxiety disorder, major depressive disorder, panic disorder, post-traumatic stress disorder, social anxiety disorder, specific phobia |
| Nasir et. Al., 2018 | Alcohol abuse, alcohol dependence, any anxiety disorder, any mental disorder, any mood disorder, any substance use disorder, bipolar disorder, dysthymia, generalised anxiety disorder, major depressive disorder, obsessive-compulsive disorder, panic disorder, post-traumatic stress disorder, social anxiety disorder, specific phobia, substance abuse, substance dependence |
| Pulay et. Al., 2009 | Schizotypal personality disorder |
| Robin et. Al., 1997 | Post-traumatic stress disorder |
| Robin et. Al., 2007 | Schizophrenia |
| Saha et. Al., 2016 | Prescription opioid use disorder |
| Sahota et. Al., 2008 | Major depressive disorder |
| Sawchuk et. Al., 2005 | Major depressive disorder, post-traumatic stress disorder |
| Shen et. Al, 2018 | Any anxiety disorder, bipolar disorder, major depressive disorder, schizophrenia |
| Smith et. Al., 2006 | Alcohol abuse, alcohol dependence, any alcohol use disorder, any anxiety disorder, any mood disorder, any substance use disorder, dysthymia, generalised anxiety disorder, major depressive disorder, mania, panic disorder, social anxiety disorder, specific phobia, substance abuse, substance dependence |
| Stinson et. Al., 2006 | Any cannabis use disorder, cannabis abuse, cannabis dependence |
| Stinson et. Al., 2008 | Narcissistic personality disorder |
| Weatherall et. Al., 2022 | Alcohol dependence |
| Zhao et. Al., 2016 | Major depressive disorder |

### Quality appraisal of included studies (JBI checklist)

| Study | Appropriate sample frame? | Appropriate sampling? | Adequate sample size? | Detailed description of subjects and setting? | Sufficient coverage of identified sample in analysis? | Valid methods used for identification of MSD? | MSD measured in standard reliable way? | Appropriate statistical analysis? | Adequate response rate? | Number of items with “yes” response | Quality appraisal |
| --- | --- | --- | --- | --- | --- | --- | --- | --- | --- | --- | --- |
| Almeida et. Al., 2014 | Yes | Yes | Unclear | Yes | Yes | Yes | Yes | Yes | Yes | 8 | High |
| Basit et Al, 2024 | Yes | Yes | Yes | Yes | Yes | Yes | Yes | Yes | Yes | 9 | High |
| Baxter et. Al., 2006 | Yes | Yes | Yes | Yes | Yes | Yes | Yes | Yes | Yes | 9 | High |
| Beals et. Al., 2005a | Yes | Yes | Yes | Yes | Yes | Yes | Yes | Yes | Yes | 9 | High |
| Beals et. Al., 2005b | Yes | Yes | Yes | Yes | Yes | Yes | Yes | Yes | Yes | 9 | High |
| Beals et. Al., 2013 | Yes | Yes | Yes | Yes | Yes | Yes | Yes | Yes | Yes | 9 | High |
| Blanco et Al., 2017 | Yes | Yes | Yes | Yes | Yes | Yes | Yes | Yes | Yes | 9 | High |
| Bohanna et. Al., 2012 | No | Unclear | Unclear | Yes | Unclear | Yes | Unclear | Yes | Unclear | 3 | Low |
| Brave Heart et. Al., 2016 | Yes | Yes | Yes | Yes | Yes | Yes | Yes | Yes | Yes | 9 | High |
| Burt et. Al., 2020 | Yes | Yes | No | Yes | Yes | Yes | Yes | Yes | Yes | 8 | High |
| Curyto et. Al., 1998 | Yes | Yes | Yes | Yes | Yes | Yes | Yes | Yes | Yes | 9 | High |
| Dashputre et. Al., 2023 | Yes | Yes | Yes | Yes | Yes | Yes | Yes | Yes | Yes | 9 | High |
| Dillard et. Al., 2012 | Yes | Yes | Yes | Yes | Yes | Yes | Yes | Yes | Yes | 9 | High |
| Emerson et. Al., 2017 | Yes | Yes | Yes | Yes | Yes | Yes | Yes | Yes | Yes | 9 | High |
| Ferrari et. Al., 2023 | Yes | Yes | Yes | Yes | Yes | Yes | Yes | Yes | Yes | 9 | High |
| Froese et. Al, 2008 | Yes | Yes | Yes | Yes | Yes | Yes | Yes | Yes | Yes | 9 | High |
| Fuller-Thomson et. Al., 2020 | Yes | Yes | Yes | Yes | Yes | Yes | Yes | Yes | Yes | 9 | High |
| Goldstein et. Al., 2016 | Yes | Yes | Yes | Yes | Yes | Yes | Yes | Yes | Yes | 9 | High |
| Grant et. Al., 2004 | Yes | Yes | Yes | Yes | Yes | Yes | Yes | Yes | Yes | 9 | High |
| Grant et. Al., 2005a | Yes | Yes | Yes | Yes | Yes | Yes | Yes | Yes | Yes | 9 | High |
| Grant et. Al., 2005b | Yes | Yes | Yes | Yes | Yes | Yes | Yes | Yes | Yes | 9 | High |
| Grant et. Al., 2006 | Yes | Yes | Yes | Yes | Yes | Yes | Yes | Yes | Yes | 9 | High |
| Grant et. Al., 2008 | Yes | Yes | Yes | Yes | Yes | Yes | Yes | Yes | Yes | 9 | High |
| Grant et. Al., 2015 | Yes | Yes | Yes | Yes | Yes | Yes | Yes | Yes | Yes | 9 | High |
| Grant et. Al., 2016 | Yes | Yes | Yes | Yes | Yes | Yes | Yes | Yes | Yes | 9 | High |
| Hasin et. Al., 2005 | Yes | Yes | Yes | Yes | Yes | Yes | Yes | Yes | Yes | 9 | High |
| Hasin et. Al., 2007 | Yes | Yes | Yes | Yes | Yes | Yes | Yes | Yes | Yes | 9 | High |
| Hasin et. Al., 2016 | Yes | Yes | Yes | Yes | Yes | Yes | Yes | Yes | Yes | 9 | High |
| Hasin et. Al., 2018 | Yes | Yes | Yes | Yes | Yes | Yes | Yes | Yes | Yes | 9 | High |
| Huang et. Al., 2006 | Yes | Yes | Yes | Yes | Yes | Yes | Yes | Yes | Yes | 9 | High |
| Kerridge et. Al., 2015 | Yes | Yes | Yes | Yes | Yes | Yes | Yes | Yes | Yes | 9 | High |
| Kerridge et. Al., 2018 | Yes | Yes | Yes | Yes | Yes | Yes | Yes | Yes | Yes | 9 | High |
| Kinzie et. Al., 1992 | Yes | Yes | Yes | Yes | Yes | Yes | Yes | Yes | Yes | 9 | High |
| Koss et. Al., 2003 | Yes | Yes | Yes | Yes | Yes | Yes | Yes | Yes | Yes | 9 | High |
| MacMillan et. Al., 2008 | Yes | Yes | Yes | Yes | Yes | Yes | Yes | Yes | Yes | 9 | High |
| Muckle et. Al., 2017 | Yes | Yes | Yes | Yes | Yes | Yes | Yes | Yes | Yes | 9 | High |
| Nadew et. Al., 2012 | No | Unclear | Unclear | Yes | Unclear | Yes | Unclear | Yes | Unclear | 3 | Low |
| Nasir et. Al., 2018 | Yes | Yes | Yes | Yes | Yes | Yes | Yes | Yes | Yes | 9 | High |
| Pulay et. Al., 2009 | Yes | Yes | Yes | Yes | Yes | Yes | Yes | Yes | Yes | 9 | High |
| Robin et. Al., 1997 | Yes | Yes | Yes | Yes | Yes | Yes | Yes | Yes | Yes | 9 | High |
| Robin et. Al., 2007 | Yes | Yes | Yes | Yes | Yes | Yes | Yes | Yes | Yes | 9 | High |
| Saha et. Al., 2016 | Yes | Yes | Yes | Yes | Yes | Yes | Yes | Yes | Yes | 9 | High |
| Sahota et. Al., 2008 | Yes | Yes | Yes | Yes | Yes | Yes | Yes | Yes | Yes | 9 | High |
| Sawchuk et. Al., 2005 | Yes | Yes | Yes | Yes | Yes | Yes | Yes | Yes | Yes | 9 | High |
| Shen et. Al, 2018 | Yes | Yes | Yes | Yes | Yes | Yes | Yes | Yes | Yes | 9 | High |
| Smith et. Al., 2006 | Yes | Yes | Yes | Yes | Yes | Yes | Yes | Yes | Yes | 9 | High |
| Stinson et. Al., 2006 | Yes | Yes | Yes | Yes | Yes | Yes | Yes | Yes | Yes | 9 | High |
| Stinson et. Al., 2008 | Yes | Yes | Yes | Yes | Yes | Yes | Yes | Yes | Yes | 9 | High |
| Weatherall et. Al., 2022 | Yes | Yes | Yes | Yes | Yes | Yes | Yes | Yes | Yes | 9 | High |
| Zhao et. Al., 2016 | Unclear | Yes | Yes | Yes | Yes | Yes | Yes | Yes | Yes | 8 | High |

### Gender specific prevalence estimates for MSD’s using diagnostic data across Australia, New Zealand, Canada and the United States of America

**Female**

|  |  | **Australia** | | | **New Zealand** | | | **Canada** | | | **United States of America** | | |
| --- | --- | --- | --- | --- | --- | --- | --- | --- | --- | --- | --- | --- | --- |
| **Mental Disorders** | **Prevalence Type** | Prevalence (%) | CI (95%) | Number of studies | Prevalence (%) | CI (95%) | Number of studies | Prevalence (%) | CI (95%) | Number of studies | Prevalence (%) | CI (95%) | Number of studies |
| Any mental disorder | Point | 43.9 | 38.5 - 49.4 | 1 | - | - | - | - | - | - | - | - | - |
|  | 12-month | 42.4 - 49.0 | 36.5 - 48.5 43.5 - 54.4 | 2 | - | - | - | - | - | - | 17.2 - 22.4 | 13.9 - 21.0 18.7 - 26.7 | 1 (2 datapoints) |
|  | Lifetime | 63.2 | 57.8 - 68.3 | 1 | - | - | - | - | - | - | 35.7 - 41.9 | 31.3 - 40.3 37.3 - 46.6 | 1 (2 datapoints) |
| Any mood disorder | Point | 18.7 | 14.6 - 23.2 | 1 | - | - | - | - | - | - | - | - | - |
|  | 12-month | 22.3 | 17.9 - 27.1 | 1 | - | - | - | - | - | - | 16.2 | 12.7 -20.3 | 1 |
|  | Lifetime | 38.9 | 33.6 - 44.3 | 1 | - | - | - | - | - | - | 33.6 | 28.9 - 38.5 | 1 |
| Any depressive disorder | Point | 10.4 | 5.2 - 15.6 | 1 | - | - | - | - | - | - | - | - | - |
|  | 12-month | - | - | - | - | - | - | - | - | - | 6.0 - 8.1 | 4.1 - 8.7 5.8 - 11.0 | 1 (2 datapoints) |
|  | Lifetime | - | - | - | - | - | - | - | - | - | 9.3 - 13.0 | 6.9 - 12.5 10.2 - 16.4 | 1 (2 datapoints) |
| Major Depressive Disorder | Point | 10.9 | 7.8 - 14.8 | 1 | - | - | - | - | - | - | 6.3- | 2.2 – 10.4 | 1 |
|  | 12-month | 13.9 - 25.8 | 10.4 - 18.1 20.8 - 31.4 | 1 | - | - | - | 18.2 | 15.5 - 21.1 | 1 | 7.3 | 5.2 - 9.8 | 2 (3 datapoints) |
|  | Lifetime | 27.3 | 22.6 - 32.3 | 1 | - | - | - | - | - | - | 13.8 | 7.7 - 21.5 | 2 (3 datapoints) |
| Dysthymia | Point | 3.7 | 1.6 - 5.7 | 1 | - | - | - | - | - | - | - | - | - |
|  | 12-month | 3.3 | 1.6 - 5.7 | 1 | - | - | - | - | - | - | 2.1 | 1.1 - 3.3 | 2 (3 datapoints) |
|  | Lifetime | 3.6 | 1.8 - 6.1 | 1 | - | - | - | - | - | - | 4.3 | 2.5 - 6.5 | 2 (3 datapoints) |
| Bipolar Disorder | Point | 2.4 | 1.0 - 4.6 | 1 | - | - | - | - | - | - | - | - | - |
|  | 12-month | 2.4 | 1.0 - 4.6 | 1 | - | - | - | - | - | - | 4.7 | 2.8 - 7.3 | 1 |
|  | Lifetime | 3.3 | 1.6 - 5.7 | 1 | - | - | - | - | - | - | 2.8 - 9.3 | 0.0 – 5.6 6.6 - 12.6 | 2 |
| Any anxiety disorder | Point | 36.5 | 31.3 - 41.8 | 1 | - | - | - | - | - | - | - | - | - |
|  | 12-month | 39.5 | 34.2 - 44.9 | 1 | - | - | - | - | - | - | 11.7 | 6.6 - 18.0 | 2 (3 datapoints) |
|  | Lifetime | 49.0 | 43.5 - 54.4 | 1 | - | - | - | - | - | - | 24.4 | 19.4 - 29.9 | 2 (3 datapoints) |
| Generalised Anxiety Disorder | Point | 5.3 | 3.2 - 8.3 | 1 | - | - | - | - | - | - | - | - | - |
|  | 12-month | 5.9 | 3.6 - 9.0 | 1 | - | - | - | - | - | - | 2.2 | 1.1 - 3.6 | 2 (3 datapoints) |
|  | Lifetime | 5.9 - 8.7 | 3.6 - 9.0 5.7 - 12.8 | 2 | - | - | - | - | - | - | 3.9 | 1.4 - 7.6 | 2 (3 datapoints) |
| Panic Disorder | Point | 7.4 | 4.8 - 10.7 | 1 | - | - | - | - | - | - | 1.1 | 0.0 – 3.0 | 1 |
|  | 12-month | 9.8 | 6.8 - 13.4 | 1 | - | - | - | - | - | - | 3.4 | 1.5 - 5.9 | 2 (3 datapoints) |
|  | Lifetime | 15.1 | 11.4 - 19.4 | 1 | - | - | - | - | - | - | 6.4 | 1.8 - 13.2 | 2 (3 datapoints) |
| Post-Traumatic Stress Disorder | Point | 17.2 | 11.3 - 21.6 | 1 | - | - | - | - | - | - | 6.2 | 2.7 - 11.8 | 1 |
|  | 12-month | 18.9 - 21.3 | 14.9 - 23.5 16.7 - 26.8 | 2 | - | - | - | - | - | - | 5.7 - 7.0 | 3.8 - 8.4 4.9 - 9.9 | 1 (2 datapoints) |
|  | Lifetime | 25.2 | 20.6 - 30.2 | 1 | - | - | - | - | - | - | 21.7 | 19.5 - 24.0 | 2 (3 datapoints) |
| Social anxiety disorder | Point | 8.6 | 5.8 - 12.1 | 1 | - | - | - | - | - | - | - | - | - |
|  | 12-month | 9.5 | 6.5 - 13.1 | 1 | - | - | - | - | - | - | 5.1 | 3.2 - 7.8 | 1 |
|  | Lifetime | 12.2 | 8.8 - 16.1 | 1 | - | - | - | - | - | - | 10.7 | 7.9 - 14.4 | 1 |
| Specific Phobia | Point | 10.9 | 7.8 - 14.8 | 1 | - | - | - | - | - | - | 1.1 | 0.0 – 3.0 | 1 |
|  | 12-month | 10.1 | 8.1 - 15.1 | 1 | - | - | - | - | - | - | 10.4 | 7.5 - 13.8 | 1 |
|  | Lifetime | 13.6 | 10.1 - 17.7 | 1 | - | - | - | - | - | - | 1.1 - 15.7 | 0.0 – 3.0 12.3 - 19.8 | 2 |
| Agoraphobia | Lifetime | - | - | - | - | - | - | - | - | - | 1.7 | 0.0 – 4.0 | 1 |
| **Substance Use Disorders** |  |  |  |  |  |  |  |  |  |  |  |  |  |
| Any substance use disorder | 12-month | 13.1 | 9.6 - 17.1 | 1 | - | - | - | - | - | - | 4.0 - 13.1 | 2.5 - 6.3 10.2 - 16.7 | 1 (2 datapoints) |
|  | Lifetime | 33.5 | 28.5 - 38.8 | 1 | - | - | - | - | - | - | 14.9 - 31.0 | 11.8 - 18.5 26.8 - 35.6 | 1 (2 datapoints) |
| Substance Abuse | Point | 1.5 | 0.4 - 3.4 | 1 | - | - | - | - | - | - | - | - | - |
|  | 12-month | 2.1 | 0.8 - 4.2 | 1 | - | - | - | - | - | - | 1.1 - 1.7 | 0.5 - 2.8 0.8 - 3.7 | 1 (2 datapoints) |
|  | Lifetime | 4.2 | 2.2 - 6.8 | 1 | - | - | - | - | - | - | 2.8 - 6.1 | 1.6 - 6.9 4.1 -8.9 | 1 (2 datapoints) |
| Substance Dependence | 12-month | 3.3 | 1.6 - 5.7 | 1 | - | - | - | - | - | - | 1.1 - 3.0 | 0.4 - 2.7 1.7 - 5.1 | 1 (2 datapoints) |
|  | Lifetime | 8.0 | 5.3 - 11.4 | 1 | - | - | - | - | - | - | 2.5 - 4.8 | 1.4 - 4.5 3.1 - 7.4 | 1 (2 datapoints) |
| Any alcohol use disorder | Point | 3.7 | 1.6 - 5.7 | 1 | - | - | - | - | - | - | 7.0 | 2.6 – 11.4 | 1 |
|  | 12-month | - | - | - | - | - | - | - | - | - | 8.7 | 6.2 - 12.1 | 1 |
|  | Lifetime | - | - | - | - | - | - | - | - | - | 32.3 – 39.4 | 27.7 - 37.2 31.0 – 47.8 | 2 |
| Alcohol Abuse | Point | 4.5 | 2.5 - 7.2 | 1 | - | - | - | - | - | - | - | - | - |
|  | 12-month | 4.7 | 2.7 - 7.6 | 1 | - | - | - | - | - | - | 3.2 | 1.2 - 6.2 | 2 (3 datapoints) |
|  | Lifetime | 15.4 | 11.7 - 19.7 | 1 | - | - | - | - | - | - | 10.7 | 5.3 - 17.6 | 3 (4 datapoints) |
| Alcohol Dependence | Point |  |  |  |  |  |  |  |  |  | 6.2 | 2.1 – 10.3 | 1 |
|  | 12-month | 6.8 | 4.3 - 10 | 1 | - | - | - | - | - | - | 3.3 | 0.4 - 8.4 | 2 (3 datapoints) |
|  | Lifetime | 19.6 | 15.4 - 24.2 | 1 | - | - | - | - | - | - | 11.3 | 5.3 - 19.1 | 3 (4 datapoints) |
| Any cannabis use disorder | 12-month | - | - | - | - | - | - | - | - | - | 5.7 | 3.3 - 8.9 | 1 |
| Opioid Use Disorder | 12-month | - | - | - | - | - | - | - | - | - | 0.5 | 0.0 - 2.4 | 1 |
|  | Lifetime | - | - | - | - | - | - | - | - | - | 2.0 | 0.7 - 4.3 | 1 |
| Nicotine Dependence | 12-month | - | - | - | - | - | - | - | - | - | 20.2 | 16.3 -24.5 | 1 |
|  | Lifetime | - | - | - | - | - | - | - | - | - | 25.2 | 21.1 - 29.9 | 1 |
| **Personality Disorders** |  |  |  |  |  |  |  |  |  |  |  |  |  |
| Any personality disorder | Lifetime | - | - | - | - | - | - | - | - | - | 21.0 | 16.9 - 25.3 | 1 |
| Avoidant personality disorder | Lifetime | - | - | - | - | - | - | - | - | - | 4.5 | 2.6 - 6.9 | 1 |
| Dependent personality disorder | Lifetime | - | - | - | - | - | - | - | - | - | 0.6 | 0.0 - 1.8 | 1 |
| Histrionic personality disorder | Lifetime | - | - | - | - | - | - | - | - | - | 2.5 | 1.3 - 4.7 | 1 |
| Obsessive-compulsive personality disorder | Lifetime | - | - | - | - | - | - | - | - | - | 8.9 | 6.2 - 12.1 | 1 |
| Paranoid personality disorder | Lifetime | - | - | - | - | - | - | - | - | - | 10.0 | 7.3 - 13.5 | 1 |
| Schizoid personality disorder | Lifetime | - | - | - | - | - | - | - | - | - | 4.6 | 2.8 - 7.3 | 1 |
| Borderline personality disorder | Lifetime |  |  |  |  |  |  |  |  |  | 10.7 | 8.4 – 13 | 1 |
| Schizotypal personality disorder | Lifetime |  |  |  |  |  |  |  |  |  | 5.8 | 4.1 – 7.5 | 1 |
| Narcissistic personality disorder | Lifetime |  |  |  |  |  |  |  |  |  | 5.2 | 3.6 – 6.8 | 1 |

*Note: Only available estimates are provided. Estimates are presented as pooled prevalence estimates (where there were three or more estimates reported), ranges (where there were two estimates reported), and single estimates (where only one estimate was available).*

**Male**

|  |  | **Australia** | | | **New Zealand** | | | **Canada** | | | **United States of America** | | |
| --- | --- | --- | --- | --- | --- | --- | --- | --- | --- | --- | --- | --- | --- |
| **Mental Disorders** | **Prevalence Type** | Prevalence (%) | CI (95%) | Number of studies | Prevalence (%) | CI (95%) | Number of studies | Prevalence (%) | CI (95%) | Number of studies | Prevalence (%) | CI (95%) | Number of studies |
| Any mental disorder | Point | 47.8 | 40.8 - 54.8 | 1 | - | - | - | - | - | - | - | - | - |
|  | 12-month | 35.1 - 56.0 | 26.1 - 45.4 48.9 - 62.9 | 2 | - | - | - | - | - | - | 25.9 - 26.1 | 21.4 -30.9 22.1 - 30.7 | 1 (2 datapoints) |
|  | Lifetime | 72.9 | 66.3 - 78.8 | 1 | - | - | - | - | - | - | 47.1 - 50 | 42.2 - 52.0 44.6 - 55.4 | 1 (2 datapoints) |
| Any mood disorder | Point | 16.9 | 12.0 - 22.7 | 1 | - | - | - | - | - | - | - | - | - |
|  | 12-month | 20.3 | 15.0 - 26.4 | 1 | - | - | - | - | - | - | 11.9 | 8.4 - 15.9 | 1 |
|  | Lifetime | 31.9 | 25.5 - 38.7 | 1 | - | - | - | - | - | - | 22.5 | 18.1 - 27.6 | 1 |
| Any depressive disorder | Point | 4 | 0.1 - 7.8 | 1 | - | - | - | - | - | - | - | - | - |
|  | 12-month | - | - | - | - | - | - | - | - | - | 3.2 - 6.3 | 2.0 - 5.3 4.1 -9.5 | 1 (2 datapoints) |
|  | Lifetime | - | - | - | - | - | - | - | - | - | 6.8 - 8.9 | 4.8 - 9.7 6.3 -12.5 | 1 (2 datapoints) |
| Major Depressive Disorder | Point | 9.2 | 5.6 - 13.9 | 1 | - | - | - | - | - | - | 2.2 | 0.0 – 4.7 | 1 |
|  | 12-month | 11.6 - 22.0 | 7.5 - 16.7 14.8 - 31.3 | 2 | - | - | - | - | - | - | 5.0 | 2.6 - 8.0 | 2 (3 datapoints) |
|  | Lifetime | 18.4 | 13.3 - 24.3 | 1 | - | - | - | - | - | - | 9.5 | 5.6 - 14.2 | 2 (3 datapoints) |
| Dysthymia | Point | 1.9 | 0.5 - 4.8 | 1 | - | - | - | - | - | - | - | - | - |
|  | 12-month | 1.9 | 0.5 - 4.8 | 1 | - | - | - | - | - | - | 0.9 | 0.2 - 2.3 | 2 (3 datapoints) |
|  | Lifetime | 2.4 | 0.7 - 5.5 | 1 | - | - | - | - | - | - | 2.5 | 1.6 - 3.6 | 2 (3 datapoints) |
| Bipolar Disorder | Point | 0.5 | 0 - 2.6 | 1 | - | - | - | - | - | - | - | - | - |
|  | 12-month | 0.5 | 0 - 2.6 | 1 | - | - | - | - | - | - | 4.3 | 2.2 - 6.9 | 1 |
|  | Lifetime | 0.9 | 0.1 - 3.4 | 1 | - | - | - | - | - | - | 7.0 | 4.4 - 10.4 | 1 |
| Any anxiety disorder | Point | 22.7 | 17.1 - 29 | 1 | - | - | - | - | - | - | - | - | - |
|  | 12-month | 25.6 | 19.8 - 32.1 | 1 | - | - | - | - | - | - | 6.7 | 4.3 - 9.6 | 2 (3 datapoints) |
|  | Lifetime | 32.9 | 26.5 - 39.7 | 1 | - | - | - | - | - | - | 14.1 | 10.2 - 18.5 | 2 (3 datapoints) |
| Generalised Anxiety Disorder | Point | 5.3 | 2.6 - 9.3 | 1 | - | - | - | - | - | - | - | - | - |
|  | 12-month | 5.8 - 6.6 | 3.0 - 9.9 3.0 - 13.6 | 2 | - | - | - | - | - | - | 0.9 | 0.5 - 1.4 | 2 (3 datapoints) |
|  | Lifetime | 5.7 | 3 - 9.91 | 1 | - | - | - | - | - | - | 2.5 | 1.2 - 4.3 | 2 (3 datapoints) |
| Panic Disorder | Point | 3.4 | 1.3 - 6.8 | 1 | - | - | - | - | - | - | - | - | - |
|  | 12-month | 6.3 | 3.3 - 10.5 | 1 | - | - | - | - | - | - | 1.3 | 0.3 - 2.8 | 2 (3 datapoints) |
|  | Lifetime | 7.2 | 4.1 - 11.6 | 1 | - | - | - | - | - | - | 2.9 | 1.5 - 4.6 | 2 (3 datapoints) |
| Post-Traumatic Stress Disorder | Point | 8.2 | 4.8 - 12.8 | 1 | - | - | - | - | - | - | 4.5 - 5.1 | 0.9 – 8.1 1.9 -10.8 | 2 |
|  | 12-month | 9.2 - 16.6 | 5.6 - 13.9 10.3 - 25.7 | 2 | - | - | - | - | - | - | 3.7 - 4.5 | 2.1 - 6.4 2.9 - 7.0 | 1 (2 datapoints) |
|  | Lifetime | 13.5 | 9.1 - 18.9 | 1 | - | - | - | - | - | - | 12.9 | 10.5 - 15.5 | 2 (3 datapoints) |
| Social anxiety disorder | Point | 6.3 | 3.3 - 10.5 | 1 | - | - | - | - | - | - | - | - | - |
|  | 12-month | 6.3 | 3.3 - 10.5 | 1 | - | - | - | - | - | - | 1.8 | 0.7 - 4.1 | 1 |
|  | Lifetime | 9.7 | 6 - 14.5 | 1 | - | - | - | - | - | - | 6.3 | 3.9 - 9.7 | 1 |
| Specific Phobia | Point | 8.2 | 4.8 - 12.8 | 1 | - | - | - | - | - | - | - | - | - |
|  | 12-month | 8.2 | 4.8 - 12.8 | 1 | - | - | - | - | - | - | 5.7 | 3.4 - 8.9 | 1 |
|  | Lifetime | 9.7 | 6 - 14.5 | 1 | - | - | - | - | - | - | 7.9 | 5.2 - 11.5 | 1 |
| **Substance Use Disorders** |  |  |  |  |  |  |  |  |  |  |  |  |  |
| Any substance use disorder | Point | 20.3 | 15 - 26.4 | 1 | - | - | - | - | - | - | - | - | - |
|  | 12-month | 28 | 22 - 34.6 | 1 | - | - | - | - | - | - | 18.9 - 22.0 | 15.0 - 23.5 18.2 - 26.4 | 1 (2 datapoints) |
|  | Lifetime | 54.6 | 47.5 - 61.5 | 1 | - | - | - | - | - | - | 42.9 - 43.1 | 37.7 - 48.4 38.3 - 48.1 | 1 (2 datapoints) |
| Substance Abuse | Point | 2.9 | 1 - 6.2 | 1 | - | - | - | - | - | - | 2.2 | 0.0 – 4.7 | 1 |
|  | 12-month | 5.3 | 2.6 - 9.3 | 1 | - | - | - | - | - | - | 2.2 - 4.5 | 1.1 - 4.4  2.9 - 7.0 | 1 (2 datapoints) |
|  | Lifetime | 15.9 | 11.2 - 2.6 | 1 | - | - | - | - | - | - | 8.1 - 10.7 | 5.6 - 11.7 8.0 -14.1 | 1 (2 datapoints) |
| Substance Dependence | Point | 4.3 | 2 - 8 | 1 | - | - | - | - | - | - | - | - | - |
|  | 12-month | 7.3 | 4.1 - 11.6 | 1 | - | - | - | - | - | - | 2.6 - 3.3 | 1.4 - 4.8 1.8 - 5.9 | 1 (2 datapoints) |
|  | Lifetime | 16.4 | 11.6 - 22.1 | 1 | - | - | - | - | - | - | 4.8 - 5.9 | 3.0 - 7.5 3..7 - 9.0 | 1 (2 datapoints) |
| Any alcohol use disorder | Point | - | - | - | - | - | - | - | - | - | 36.4 | 28.2 – 44.6 | 1 |
|  | 12-month | - | - | - | - | - | - | - | - | - | 15.9 | 12.1 - 20.5 | 1 |
|  | Lifetime | - | - | - | - | - | - | - | - | - | 54.7 – 76.5 | 49.1 - 60.4 69.3 – 83.7 | 2 |
| Alcohol Abuse | Point | 6.3 | 3.3 - 10.5 | 1 | - | - | - | - | - | - | 3.6 | 0.4 – 6.8 | 1 |
|  | 12-month | 11.6 | 7.5 - 16.7 | 1 | - | - | - | - | - | - | 7.8 | 6.6 - 9.1 | 2 (3 datapoints) |
|  | Lifetime | 25.6 | 19.8 - 32.1 | 1 | - | - | - | - | - | - | 18.9 | 11.5 - 2.77 | 3 (4 datapoints) |
| Alcohol Dependence | Point | 10.1 | 6.3 - 15.1 | 1 | - | - | - | - | - | - | 32.8 | 24.8 – 40.8 | 1 |
|  | 12-month | 15.5 | 10.8 - 21.1 | 1 | - | - | - | - | - | - | 10.1 | 7.5 - 13.0 | 2 (3 datapoints) |
|  | Lifetime | 28.5 | 22.4 - 35.1 | 1 | - | - | - | - | - | - | 23.1 | 17.4 - 29.3 | 3 (4 datapoints) |
| Any cannabis use disorder | 12-month | - | - | - | - | - | - | - | - | - | 4.8 | 2.3 - 8.5 | 1 |
| Opioid Use Disorder | 12-month | - | - | - | - | - | - | - | - | - | 2.8 | 1.1 - 6.1 | 1 |
|  | Lifetime | - | - | - | - | - | - | - | - | - | 6.1 | 3.3 - 10.4 | 1 |
| Nicotine Dependence | 12-month | - | - | - | - | - | - | - | - | - | 26.4 | 21.6 - 31.7 | 1 |
|  | Lifetime | - | - | - | - | - | - | - | - | - | 35.9 | 30.7 - 41.6 | 1 |
| **Personality Disorders** |  |  |  |  |  |  |  |  |  |  |  |  |  |
| Any personality disorder | Lifetime | - | - | - | - | - | - | - | - | - | 27.6 | 22.8 - 33.0 | 1 |
| Avoidant personality disorder | Lifetime | - | - | - | - | - | - | - | - | - | 3.0 | 1.3 - 5.4 | 1 |
| Dependent personality disorder | Lifetime | - | - | - | - | - | - | - | - | - | 0.4 | 0.0 - 2.3 | 1 |
| Histrionic personality disorder | Lifetime | - | - | - | - | - | - | - | - | - | 2.3 | 0.9 - 4.5 | 1 |
| Obsessive-compulsive personality disorder | Lifetime | - | - | - | - | - | - | - | - | - | 11.2 | 7.9 - 15.2 | 1 |
| Paranoid personality disorder | Lifetime | - | - | - | - | - | - | - | - | - | 11.3 | 8.2 - 15.5 | 1 |
| Schizoid personality disorder | Lifetime | - | - | - | - | - | - | - | - | - | 8.1 | 5.5 - 11.9 | 1 |
| Borderline personality disorder | Lifetime |  |  |  |  |  |  |  |  |  | 13.2 | 10.7 – 15.7 | 1 |
| Schizotypal personality disorder | Lifetime |  |  |  |  |  |  |  |  |  | 7.5 | 5.6 – 9.4 | 1 |
| Narcissistic personality disorder | Lifetime |  |  |  |  |  |  |  |  |  | 9.5 | 7.3 – 11.7 | 1 |

*Note: Only available estimates are provided. Estimates are presented as pooled prevalence estimates (where there were three or more estimates reported), ranges (where there were two estimates reported), and single estimates (where only one estimate was available).*

### Forest plots and funnel plots for meta-analyses

#### Alcohol abuse, 12-month, both

Hasin 2007

Beals 2005a

Beals 2005b

#### Alcohol abuse, 12-month, female

Braveheart 2015

Beals 2005a

Beals 2005b

#### Alcohol abuse, 12-month, male

Braveheart 2015

Beals 2005b

Beals 2005a

#### Alcohol abuse, lifetime, both

Beals 2005b

Koss 2003

Beals 2005a

Braveheart 2015

#### Alcohol abuse, lifetime, female

Koss 2003

Braveheart 2015

Beals 2005b

Beals 2005a

#### Alcohol abuse, lifetime, male

Koss 2003

Braveheart 2015

Beals 2005b

Beals 2005a

#### Alcohol dependence, 12-month, both

Hasin 2007

Beals 2005b

Beals 2005a

#### Alcohol dependence, 12-month, female

Beals 2005a

Beals 2005b

Braveheart 2015

#### Alcohol dependence, 12-month, male

Beals 2005b

Beals 2005a

Braveheart 2015

#### Alcohol dependence, lifetime, both

Koss 2003

Beals 2005a

Beals 2005b

Hasin 2007

#### Alcohol dependence, lifetime, female

Beals 2005a

Beals 2005b

Braveheart 2015

Koss 2003

#### Alcohol dependence, lifetime, male

Beals 2005b

Beals 2005a

Braveheart 2015

Koss 2003

#### Any anxiety disorder, 12-month, both

Smith 2006

Beals 2005b

Beals 2005a

#### Any anxiety disorder, 12-month, female

Braveheart 2015

Beals 2005a

Beals 2005b

#### Any anxiety disorder, 12-month, male

Beals 2005b

Beals 2005a

Braveheart 2015

#### Any anxiety disorder, lifetime, both

Braveheart 2015

Beals 2005a

Beals 2005b

#### Any anxiety disorder, lifetime, female

Beals 2005b

Beals 2005a

Braveheart 2015

#### Any anxiety disorder, lifetime, male

Braveheart 2015

Beals 2005a

Beals 2005b

#### Any substance use disorder, 12-month, both

Smith 2006

Beals 2005b

Grant 2016

Beals 2005a

#### Any substance use disorder, lifetime, both

Grant 2016

Beals 2005b

Beals 2005a

#### Dysthymia, 12-month, both

Smith 2006

Beals 2005a

Beals 2005b

#### Dysthymia, 12-month, female

Beals 2005b

Braveheart 2015

Beals 2005a

#### Dysthymia, 12-month, male

Braveheart 2015

Beals 2005a

Beals 2005b

#### Dysthymia, lifetime, both

Braveheart 2015

Beals 2005b

Beals 2005a

#### Dysthymia, lifetime, female

Beals 2005b

Beals 2005a

Braveheart 2015

#### Dysthymia, lifetime, male

Beals 2005b

Beals 2005a

Braveheart 2015

#### Generalised anxiety disorder, 12-month, both

Beals 2005b

Smith 2006

Beals 2005a

#### Generalised anxiety disorder, 12-month, male

Beals 2005b

Beals 2005a

Braveheart 2015

#### Generalised anxiety disorder, 12-month, female

Beals 2005b

Beals 2005a

Braveheart 2015

#### Generalised anxiety disorder, lifetime, both

Emerson 2017

Beals 2005a

Beals 2005b

Braveheart 2015

#### Generalised anxiety disorder, lifetime, male

Beals 2005b

Beals 2005a

Braveheart 2015

#### Generalised anxiety disorder, lifetime, female

Braveheart 2015

Beals 2005a

Beals 2005b

#### Major depressive disorder, 12-month, both

Smith 2006

Hasin 2007

Beals 2005b

Beals 2005a

#### Major depressive disorder, 12-month, female

Braveheart 2015

Beals 2005a

Beals 2005b

#### Major depressive disorder, 12-month, male

Beals 2005b

Beals 2005a

Braveheart 2015

#### Major depressive disorder, lifetime, both

Emerson 2017

Sawchuk 2005

Hasin 2007

Beals 2005b

Beals 2005a

#### Major depressive disorder, lifetime, female

Beals 2005b

Beals 2005a

Braveheart 2015

#### Major depressive disorder, lifetime, male

Beals 2005b

Beals 2005a

Braveheart 2015

#### Panic disorder, 12-month, both

Grant 2006

Beals 2005b

Beals 2005a

#### Panic disorder, 12-month, female

Braveheart 2015

Beals 2005a

Beals 2005b

#### Panic disorder, 12-month, male

Beals 2005b

Beals 2005a

Braveheart 2015

#### Panic disorder, lifetime, both

Beals 2005a

Beals 2005b

Grant 2006

#### Panic disorder, lifetime, female

Braveheart 2015

Beals 2005a

Beals 2005b

#### Panic disorder, lifetime, male

Beals 2005b

Beals 2005a

Braveheart 2015

#### Post-traumatic stress disorder, 12-month, both

Beals 2005b

Beals 2005a

Goldstein 2016

#### Post-traumatic stress disorder, lifetime, both

Robin 1997

Sawchuk 2005

Emerson 1997

Beals 2005a

Beals 2005b

#### Post-traumatic stress disorder, lifetime, male

Beals 2005b

Beals 2005a

Robin 1997

#### Post-traumatic stress disorder, lifetime, female

Robin 1997

Beals 2005b

Beals 2005a

#### Substance abuse, 12-month, both

Beals 2005a

Beals 2005b

Smith 2006

#### Substance dependence, 12-month, both

Beals 2005a

Beals 2005b

Smith 2006

#### Prescription opioid use disorder, lifetime, both

Kerridge 2015

Saha 2016

Huang 2006

#### Major depressive disorder (symptom scale), point-prevalence, both

Zhao 2016

Sahota 2008

Curtyo 1998

Dillard 2012

#### Major depressive disorder (symptom scale), point-prevalence, female

Dillard 2012

Sahota 2008

Zhao 2016

#### Major depressive disorder (symptom scale), point-prevalence, male

Zhao 2016

Sahota 2008

Dillard 2012
